# Causal effects of inflammatory bowel diseases on the risk of kidney stone disease: a two-sample bidirectional mendelian randomization

**DOI:** 10.1186/s12894-023-01332-4

**Published:** 2023-10-12

**Authors:** Huayang Zhang, Yong Huang, Junyong Zhang, Huiyi Su, Chengguo Ge

**Affiliations:** 1 Department of Urology, The Second Affiliated Hospital of Chongqing Medical University, Chongqing, 400010 China; 2 Department of Urology, The First Affiliated Hospital of Chongqing Medical University, Chongqing, 400016 China; 3 Department of Respiratory Medicine, Children’s Hospital of Chongqing Medical University, Chongqing, 400014 China

**Keywords:** Kidney stone disease, Inflammatory bowel disease, Crohn’s disease, Ulcerative colitis, Bidirectional mendelian randomization

## Abstract

**Background:**

Existing epidemiological observational studies have suggested interesting but inconsistent clinical correlations between inflammatory bowel disease (IBD), including Crohn’s disease (CD) and ulcerative colitis (UC), and kidney stone disease (KSD). Herein, we implemented a two-sample bidirectional Mendelian randomization (MR) to investigate the causal relationship between IBD and KSD.

**Methods:**

Data on IBD and KSD were obtained from Genome-Wide Association Studies (GWAS) summary statistics and the FinnGen consortium, respectively. Strict selection steps were used to screen for eligible instrumental SNPs. We applied inverse variance weighting (IVW) with the fix-effects model as the major method. Several sensitivity analyses were used to evaluate pleiotropy and heterogeneity. Causal relationships between IBD and KSD were explored in two opposite directions. Furthermore, we carried out multivariable MR (MVMR) to obtain the direct causal effects of IBD on KSD.

**Results:**

Our results demonstrated that CD could increase the risk of KSD (IVW: OR = 1.06, 95% CI = 1.03–1.10, *p* < 0.001). Similar results were found in the validation group (IVW: OR = 1.05, 95% CI = 1.01–1.08, *p* = 0.013) and in the MVMR analysis. Meanwhile, no evidence of a causal association between UC and KSD was identified. The reverse MR analysis detected no causal association.

**Conclusions:**

This MR study verified that CD plays a critical role in developing kidney stones and that the effect of UC on KSD needs to be further explored.

**Supplementary Information:**

The online version contains supplementary material available at 10.1186/s12894-023-01332-4.

## Background

Inflammatory bowel disease (IBD), including Crohn’s disease (CD) and ulcerative colitis (UC), belongs to a group of chronic diseases occurring in the gastrointestinal tract with increasing incidence across the world [1]. The number of patients suffering from IBD worldwide is expected to grow exponentially over the next few decades, which will pose a huge challenge to healthcare systems [2]. In addition to the characteristic symptoms of gastric discomfort and diarrhea, extraintestinal symptoms frequently occur in patients with IBD [3]. Studies have reported that IBD is related to various extraintestinal manifestations, containing ophthalmologic, genitourinary, dermatologic, hematologic, pulmonary, cardiovascular, neurologic, pancreatic and hepatobiliary systems [4]. However, CD and UC can affect the development of extraintestinal complications to varying degrees [5].

The incidence of kidney stone disease (KSD) increases across sex, race and age, affecting approximately 15% of the population [6]. For the majority of the population, KSD is caused by a multifactorial etiology involving genetic and environmental factors. Genetic approaches studying KSD have uncovered that the following play significant roles in the formation of renal stones: channels and transporters; ions, protons and amino acids; the metabolic pathways for cysteine, vitamin D, oxalate, uric acid and purines; and the calcium-sensitive receptor signaling pathway [7]. Extraintestinal manifestations are quite common in patients with IBD, and kidney stone disease has been reported with increasing frequency in these patients and with a higher risk of CD than UC [8, 9]. As for its relevant pathogenesis, hypercalcemia and osteoporosis due to long-term corticosteroid exposure may contribute to the development of calcium-containing calculus [10]. Furthermore, the gut microbiota residing in the human gastrointestinal tract has been reported to be involved in the formation of renal calculus [11], making the interaction between IBD and the gut microbiota a key risk factor [12]. In a large cohort study, the risk of urolithiasis was related to anti-TNF therapy and surgery. Small bowel resection or ileostomy will alter intestinal absorption in patients diagnosed with IBD [13]. Interestingly, a clinical trial showed kidney stones were discovered in none of the UC patients but in some of the CD patients. Meanwhile, hyperoxaluria occurred in 36% of patients with CD but was absent in those with UC, suggesting the occurrence of renal calculi might be related to the patients with CD [14]. Nevertheless, the genetic correlation and causal relationship between IBD and KSD remain unclear at present.

Traditional epidemiological approaches have some limitations that affect causal estimates. In observational studies, the causal link between IBD and KSD is more likely to be biased by the likelihood of potential confounders such as age [15], oxaliplatin [16], obesity [17, 18] and lipid metabolism [19, 20]. In addition to the drawbacks mentioned in observational studies, randomized controlled trials (RCTs) are identically difficult to directly investigate the etiology of diseases due to the strict control of experimental conditions, the high standards of experimental design and implementation and medical ethical considerations. Two-sample Mendelian randomization (MR) is a method that employs genetic variants as instrument variables (IVs) of exposure. It is widely applied to study the causal relationship between potential risk elements and health outcomes in observational data [21]. Besides, it can not only avoid the insurmountable problems present in epidemiological studies but also generate more reliable evidence concerning which interventions are supposed to produce health benefits [22]. In the current study, a two-sample bidirectional and multivariable MR analysis was performed to infer the causal association between IBD (including CD and UC) and KSD.

## Materials and methods

### Study design and flowchart

**Fig. 1:**
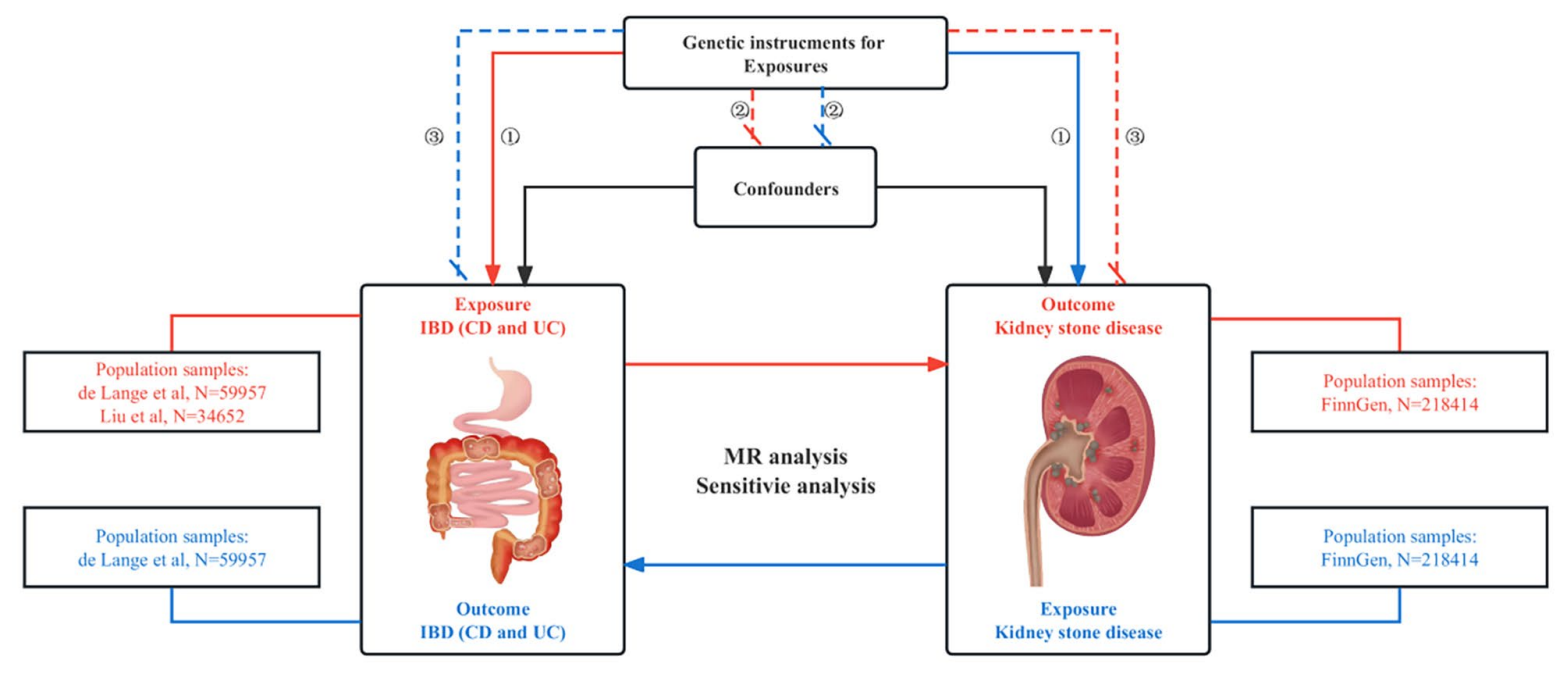
Study design overview. Notes: MR analysis follows 3 key assumptions: (I) Genetic variants applied as IVs should be robustly correlated with exposure; (II) Genetic variants applied are supposed not to link to any confounders; (III) Genetic variants selected should affect the risk of KSD only via IBD rather than other pathways. The orange line represents a Mendelian analysis of the genetic correlation between IBD (including CD and UC) and KSD; The blue line represents a Mendelian analysis of the genetic correlation between KSD and IBD (including CD and UC). Abbreviations: FinnGen, FinnGen Consortium; IBD, inflammatory bowel disease; CD, Crohn’s disease; UC, ulcerative colitis

**Fig. 2:**
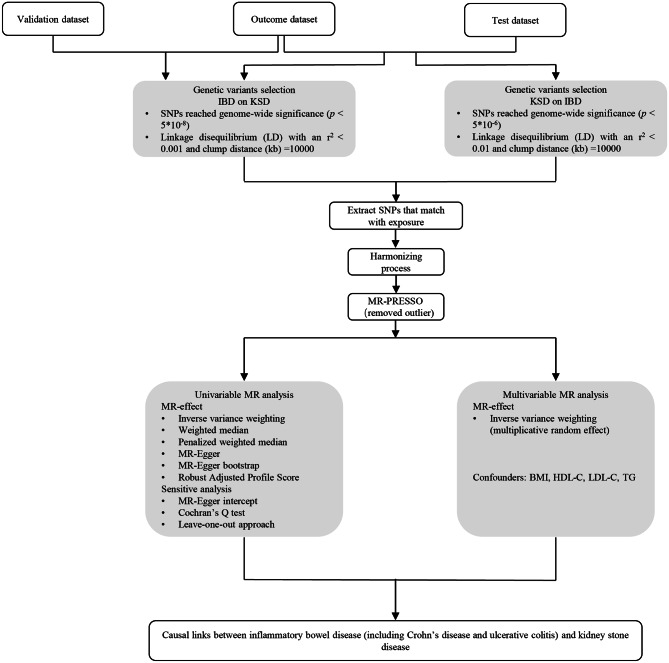
Flow chart on how to perform MR analysis step by step. Abbreviations: SNPs, single-nucleotide polymorphisms; MR-PRESSO, MR-Pleiotropy Sum and Outlier method; val, validation; IBD, inflammatory bowel disease; CD, Crohn’s disease; UC, ulcerative colitis; KSD, kidney stone disease

A two-sample bidirectional MR analysis was applied to investigate the genetic correlation between IBD (including CD and UC) and KSD. Briefly, we firstly implemented univariable MR analysis to investigate the causal effect of IBD (including CD and UC) on the risk of KSD and then conducted it in the reverse direction. Next, MVMR was further performed to assess the direct effects of IBD on KSD after adjusting for confounders. All MR analysis follows three key assumptions as below [23] (Fig. 1): (I) Genetic variants applied as IVs should be robustly correlated with exposure; (II) Genetic variants applied are supposed not to link to any confounders; (III) Genetic variants selected should affect the risk of KSD only via IBD rather than other pathways. The flowchart of the Two-sample MR analysis is shown in Fig. 2.

### Data sources for IBD and KSD

Summary GWAS data from two different sources was used for IBD. The test dataset for IBD (including CD and UC) contained a total of 59,957 individuals of predominantly European ancestry [24]. The data from this large meta-analysis GWAS comprised 25,042 IBD cases with 34,915 noncases, 12,194 CD cases with 28,072 noncases and 12,366 UC cases with 33,609 noncases, respectively. The validation dataset from the International Inflammatory Bowel Disease Genetics Consortium (IIBDGC) was based on 15 studies of the European population. It included 12,882 cases of IBD (21,770 controls), 5956 cases of CD (14,927 controls) and 6968 cases of UC (20,464 controls). All patients were diagnosed in the clinic by endoscopic, radiological and histopathological examinations [25].

Summary-level data for KSD was derived from the FinnGen consortium (https://finngen.gitbook.io/documentation/). Cases of European descent were defined by 592 in ICD-8 and ICD-9 and N20 in ICD-10. After excluding participants with ambiguous sex, high genotype deletion (> 5%), excessive heterozygosity (± 4 SD) and non-Finnish ancestry, the fifth release of the FinnGen consortium data was employed. The dataset enrolled 4969 cases with calculi of the kidney and ureter and 213,445 controls [26]. Detailed information on the data used in the MR analysis is displayed in Table 1.

**Table 1:** Detailed information on the data used in Mendelian randomization analysis

Traits	Data sources	Sample size(cases/controls)	Population	Use
IBD	de Lange et al. [24]	59,957(25,042/34,915)	European	Exposure/Outcome
CD		40,266(12,194/28,072)		
UC		45,975(12,366/33,609)		
IBD (val)	Liu et al. [25]	34,652(12,882/21,770)	European	Exposure for validation
CD (val)		20,883(5956/14,927)		
UC (val)		27,432(6968/20,464)		
KSD	FinnGen	218,414(4969/213,445)	European	Outcome/Exposure
HDL-C	UK Biobank [34]	403,943	European	Confounders for MVMR analyses
LDL-C		440,546		
TG		441,016		
BMI	Hoffmann et al. [33]	315,347	European	Confounder for MVMR analysis

IBD, inflammatory bowel disease; CD, Crohn’s disease; UC, ulcerative colitis; KSD, kidney stone disease; val, validation; HDL-C, high-density lipoprotein cholesterol; LDL-C, low-density lipoprotein cholesterol; TG, triglycerides; BMI, body mass index; MVMR, multivariable Mendelian randomization; FinnGen, FinnGen consortium; UK Biobank, UK Biobank consortium

### Genetic instrumental variants selection

We performed several steps to extract qualified instrumental single-nucleotide polymorphisms (SNPs) based on the publicly available GWAS data on IBD (including CD and UC). First, we identified SNPs highly related to the IBD with genome-wide significance (*p* < 5E-8) to meet the first assumption. Second, the significant SNPs were then clumped by linkage disequilibrium (LD) with an r^2^ < 0.001 and clump distance (kb) = 10,000 for acquiring independent instruments. Meanwhile, F statistics for each instrument were calculated with the equation F = β^2^ exposure / SE^2^ exposure to evaluate the instrument’s strength (F value < 10 were considered weak IVs) [27]. Third, some ineligible SNPs were discarded if proxies were not available. Ambiguous and palindromic variants whose effects couldn’t be corrected in the harmonizing process were also excluded. Additionally, in our analysis, outlier SNPs affecting horizontal pleiotropy will be ruled out by the MR Pleiotropy RESidual Sum and Outlier (MR-PRESSO) method [28]. In the reverse MR analysis, we adjusted the threshold (*p* < 5E-6) and LD with an r^2^ < 0.01 for obtaining more IVs contributing to KSD [29]. Detailed information on SNPs for IBD, CD, UC and KSD was presented in Additional File 1 Tables S2-S10.

### Mendelian randomization analysis

All MR analyses were conducted in the R software (version 4.2.1) with the TwoSampleMR (version 0.5.6) [30] and MRPRESSO (version 1.0) [28] packages.

### Univariable MR

We chose inverse variance weighting (IVW) as the main method for estimating the bidirectional causality of IBD (including CD and UC) and KSD [31]. For each SNP, the Wald ratio was used to generate MR impact estimates. The Wald ratios were meta-analyzed using IVW. If heterogeneity existed (*p* < 0.05), the IVW approach of the random effects model was applied, otherwise, we used the fixed-effects IVW model. Furthermore, a pooled causal meta-analysis estimate was generated for both IBD populations adopting a fixed-effects model. The relative risk caused by the exposure was evaluated using the odds ratio (OR) and corresponding 95% confidence intervals (CIs).

### Multivariable MR

Published studies have reported that obesity or body mass index (BMI) and dyslipidemia are strongly related to the occurrence of kidney stones [17, 19, 32]. Coincidentally, increasing rates of obesity and persistent changes in lipid levels have been demonstrated in patients with IBD [18, 20]. Moreover, we identified some SNPs linked to BMI, high-density lipoprotein cholesterol (HDL-C), low-density lipoprotein cholesterol (LDL-C) and triglycerides (TG) via searching for PhenoScanner (Additional File 2: Table S2). Therefore, MVMR was performed to assess the direct effects of IBD, CD and UC on KSD independent of potentially influential confounding factors. The robust IVW approach with multiplicative random effect was employed as the main approach. The relevant data involving BMI [33], HDL-C, LDL-C and TG [34] were obtained from publicly available GWAS summary statistics. Specific information for every single GWAS statistic was exhibited in Table 1.

### Sensitivity analyses

As complementary analyses, the MR-Egger [35], the weighted median (WM) [36], MR Robust Adjusted Profile Score (MR-RAPS) [37] [28], MR-Egger bootstrap and penalized weighted median approaches were implemented to evaluate the reliability and stability of the outcomes. Among these analyses, the MR-Egger regression cannot be influenced by the validity of the instrumental variables. It provides estimates after correcting for directional pleiotropic effects [35]. The WM method can generate accurate results if the weight of valid IVs surpasses 50% [36]. The MR-RAPS approach outperforms other conventional MR estimates in terms of statistical power and is robust to both systematic and idiosyncratic pleiotropy [37]. It can provide a robust inference for MR analysis by executing a linear model to adjust for the profile possibility of the summary data. This estimate takes the weak IV bias into account and remains consistent in the presence of weak IVs. Penalized weighted median is a robust MR approach that aims to estimate the causal effect by taking a weighted median of individual causal estimates from different genetic variants. It penalizes the weights of down-weighted variants with high horizontal pleiotropy or outlying causal estimates. Egger bootstrap is a modification of the Egger regression method in MR analysis. It addresses the potential bias caused by horizontal pleiotropy. Meanwhile, it uses bootstrap resampling to estimate the uncertainty around the causal effect estimates and provides confidence intervals.

Since pleiotropy in MR analyses may result in confounding and bias in MR estimations, we implemented a couple of approaches to identify possible pleiotropy. First, Cochran’s Q test was utilized to perform a heterogeneity test. Heterogeneities were identified when the p-value of the Cochran Q statistics was less than 0.05. Second, the MR-Egger intercept test was performed to analyze the potential pleiotropic impacts of SNPs serving as IVs [35]. In the MR-PRESSO approach, it consists of three functions: (i) the MR-PRESSO global test for testing horizontal pleiotropy, (ii) the MR-PRESSO distortion test for examining significant differences in estimates of causality before and after outlier correction, (iii) the MR-PRESSO outlier test for correcting horizontal pleiotropy [28]. Third, we conducted a leave-one-out analysis to identify whether the causal estimations were driven by any individual SNP with a large effect. This method removes one SNP at a time and performs IVW on the remaining SNPs. If the results vary significantly after the removal of a specific SNP, this indicates that the SNP may be a potentially influential variant, and we should draw a conclusion with caution [38]. Last, the PhenoScanner [39, 40] (version 2.0) database (http://www.phenoscanner.medschl.cam.ac.uk/) was searched to determine whether the results were affected by any potential genetic variants correlated with confounders. We set the significance threshold of the p-value at 5E-8 to determine if any SNPs were strongly associated with confounding factors [41]. The MR analysis was then conducted again after removing potentially influential SNPs.

## Results

### Univariable MR analysis

#### The causal effects of IBD on KSD

**Fig. 3:**
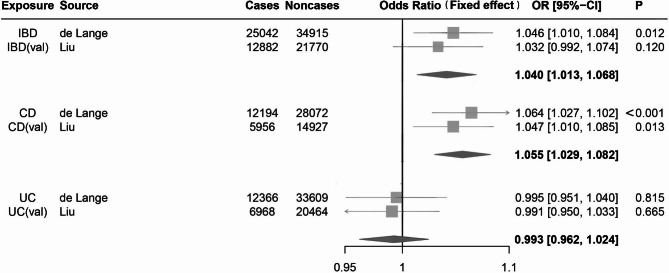
Forest plots of causal estimates for the effect of IBD, CD and UC on KSD using fix-effects IVW method. (**A**) Forest plots of causal estimates for the effect of IBD on KSD. (**B**) Forest plots of causal estimates for the effect of CD on KSD. (**C**) Forest plots of causal estimates for the effect of UC on KSD. ORs for KSD were scaled to genetically predicted IBD, CD and UC. Abbreviations: OR, odds ratio; CI, confidence interval; IBD, inflammatory bowel disease; CD, Crohn’s disease; UC, ulcerative colitis; val, validation

**Fig. 4:**
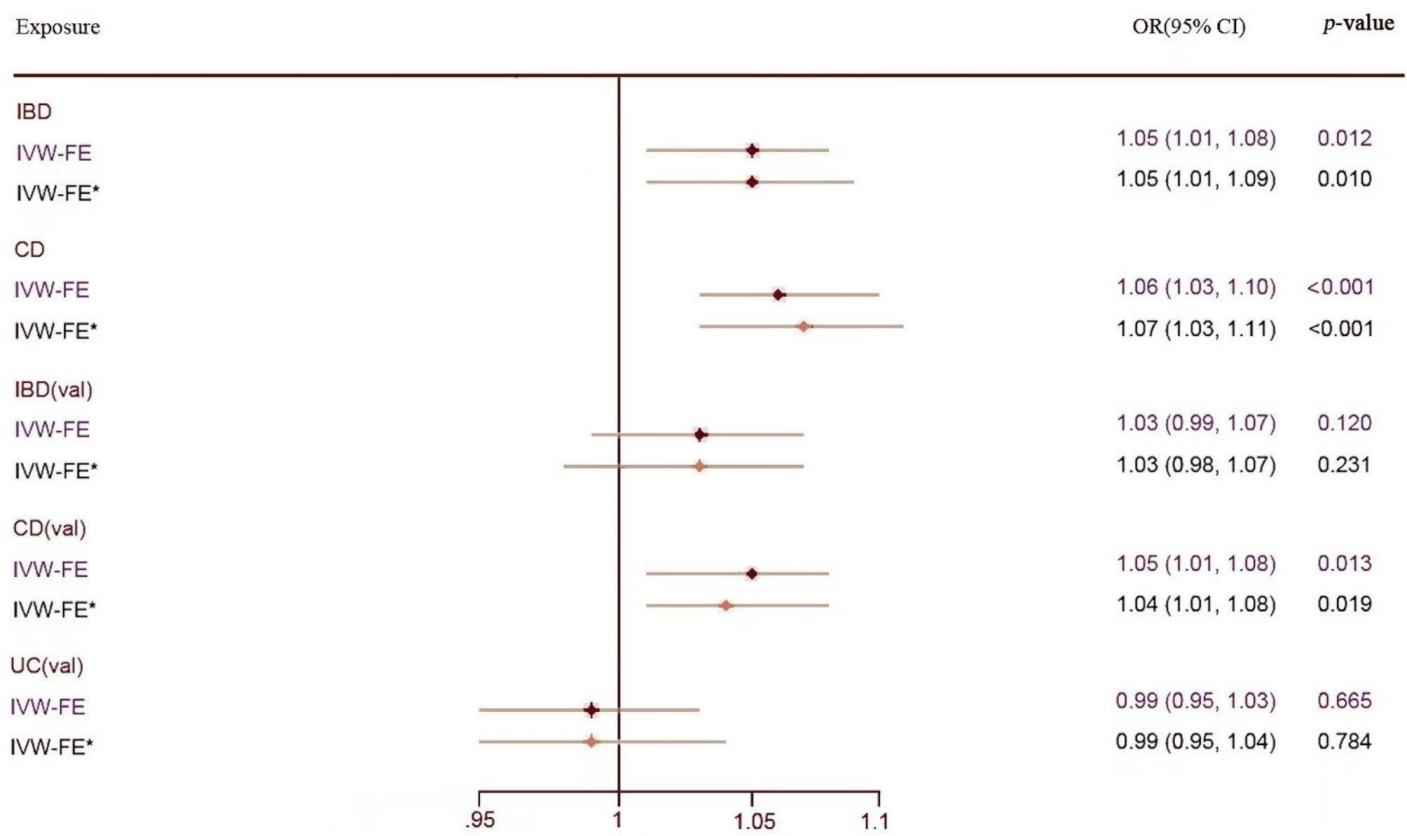
Comparison of Mendelian randomization estimates of IBD, CD and UC on KSD after removing pleiotropic genetic variants. Abbreviations: IBD, inflammatory bowel disease; CD, Crohn’s disease; UC, ulcerative colitis; val, validation; IVW-FE, inverse variance weighted with fixed effects model; OR, odds ratio; *Pleiotropic SNPs excluded

After a strict screening process, we finally included 98 SNPs for IBD in the test group (Additional File 1: Table S2) and 123 SNPs in the validation group (Additional File 1: Table S5). There were no weak instrumental variants (F statistics>10). As the Cochran Q test revealed no heterogeneity (*p*>0.05), we applied the IVW method with the fix-effect model. Genetically predicted IBD as a whole was positively related to an elevated risk of kidney stones in the test group (IVW(FE) OR = 1.05, 95% CI = 1.01–1.08, *p* = 0.012), but the validation group didn’t detect any causal relationship (IVW (FE) OR = 1.03, 95% CI = 0.99–1.07, *p* = 0.120). The MR-RAPS, WM and penalized weighted median methods showed similar results (Additional File 2: Table S1). To obtain the combined effect size, we performed a meta-analysis using the fixed-effect model. A substantial causal relationship was observed between IBD and KSD (IVW (FE) OR = 1.04, 95% CI = 1.01–1.07, *p* = 0.003, *I*^2^ = 0%) (Fig. 3). Although MR-Egger estimates and Egger bootstrap estimates were inconsistent with the IVW estimates, we still regarded the IVW estimates as the most robust evidence, as no statistically significant horizontal pleiotropy was detected (*p*-value for MR-Egger intercept > 0.05). The results were not changed after ruling out pleiotropic IVs found in the PhenoScanner (Fig. 4, Additional File 2: Table S1).

Scatter plots, funnel plots and plots of “MR-effect” analyses for MR analysis were provided in Additional File 2: Figures S1, S2 and S3. “Leave-one-out” sensitivity analysis in the test group (Additional File 2: Figure S4) presented that rs1864239 located on chromosome 15 might be a potentially influential SNP affecting the causal association between IBD and KSD. However, its related gene, ST20-MTHFS, belongs to a kind of methyltetrahydrofolate synthase that has not been proven to be involved in the metabolism of KSD according to the existing studies. Hence, we performed MR analysis by keeping this SNP and drew a cautious conclusion.

#### The causal effects of CD on KSD

According to the selection criteria, 75 SNPs for CD in the test group (Additional File 1: Table S3) and 109 SNPs in the validation group (Additional File 1: Table S6) were finally included in our analyses. With the F-statistic of IVs being much greater than 10, it indicated that the likelihood of bias caused by weak IVs was small. In this part, the MR Egger tests indicated no significant pleiotropy (*p*-value for MR-Egger intercept > 0.05), and the Cochran Q test showed no heterogeneity in the two groups (*p* > 0.05). With the fixed-effect model, the IVW method demonstrated a statistically significant causal impact of CD on KSD in both the test group (IVW (FE) OR = 1.06, 95% CI = 1.03–1.10, *p* < 0.001) and validation group (IVW (FE) OR = 1.05, 95% CI = 1.01–1.08, *p* = 0.013). Moreover, the synthetic effects shared similar results to support such a causal association (IVW (FE) OR = 1.06, 95% CI = 1.03–1.08, *p*<0.001, *I*^2^ = 0%) (Fig. 3). Our results remained consistent after removing pleiotropic IVs identified in the PhenoScanner (Fig. 4, Additional File 2: Table S1). MR-RAPS results presented a significant causal association in the test group (*p* = 0.005) but no causal relationship in the validation group (*p* = 0.068). The other MR estimates identified no statistical significance of causality (Additional File 2: Table S1). Scatter plots, funnel plots and plots of the “MR-effect” for two groups were offered in Additional File 2: Figures S1, S2 and S3. “Leave-one-out” sensitivity analysis detected no potentially influential SNPs driving the causality in two groups (Additional File 2: Figure S4), and thus, we could draw a robust conclusion.

#### The causal effects of UC on KSD

In this section, we screened out 48 SNPs for UC in the test group (Additional File 1: Table S4) and 82 SNPs in the validation group (Additional File 1: Table S7) as the effective IVs. The F statistics for IVs of UC in the two groups were likewise greater than 10. Likewise, the Cochran Q test detected no significant evidence of heterogeneity (*p*>0.05), and the MR Egger regression test identified no horizontal pleiotropy (*p*-value for MR-Egger intercept > 0.05). Consequently, we used the fixed-effects model to calculate the causal estimates. The IVW method identified no causal evidence between UC and KSD in two groups (IVW (FE) OR = 0.99, 95% CI = 0.95–1.04, *p* = 0.815; IVW (FE) OR = 0.99, 95% CI = 0.95–1.03, *p* = 0.665). After pooling the effect sizes, there was no statistical significance. (IVW (FE) OR = 0.99, 95% CI = 0.96–1.02, *p* = 0.634, *I*^2^ = 0%) (Fig. 3). Equivalent findings were confirmed by other MR estimates (Additional File 2: Table S1). Furthermore, we reached the same conclusion after excluding potentially pleiotropic IVs correlated to confounding factors (Fig. 4, Additional File 2: Table S1).

Scatter plots, funnel plots and “MR-effect” plots of the MR effect for two groups were supplied in Additional File 2: Figures S1, S2 and S3. “Leave-one-out” sensitivity analysis determined no potentially influential SNPs existed in the two groups (Additional File 2: Figure S4). In summary, genetically predicted UC had no causal effect on KSD, and our results were stable.

### The reverse MR analysis

In order to determine whether KSD exerts an inverse effect on IBD, we performed a reverse MR analysis using IVW as the primary method. In the reverse MR analyses, no causal impacts of KSD on IBD (IVW (FE) OR = 1.04, 95% CI = 1.00–1.08, *p* = 0.082) and CD (IVW (FE) OR = 1.03, 95% CI = 0.98–1.09, *p* = 0.234) were detected through the fixed-effects IVW method. Since heterogeneity and horizontal pleiotropy (Cochran’s Q test, *p* = 0.038; MR-PRESSO global test, *p* = 0.047) were observed only with UC as the outcome, we converted the fixed-effect model for this group of IVW method into a random-effect model. Consequently, no causal evidence was identified between KSD and UC (IVW (RE) OR = 1.05, 95% CI = 1.00–1.11, *p* = 0.071).

The corresponding results of the MR and sensitivity analyses are available in Additional File 2: Table S1. The “Leave-one-out” plot showed that our results were robust and stable (Additional File 2: Figure S8). In addition, scatter plots, funnel plots and “MR-effect” plots were shown in Additional File 2: Figures S5, S6 and S7.

### Multivariable MR analyses

**Fig. 5:**
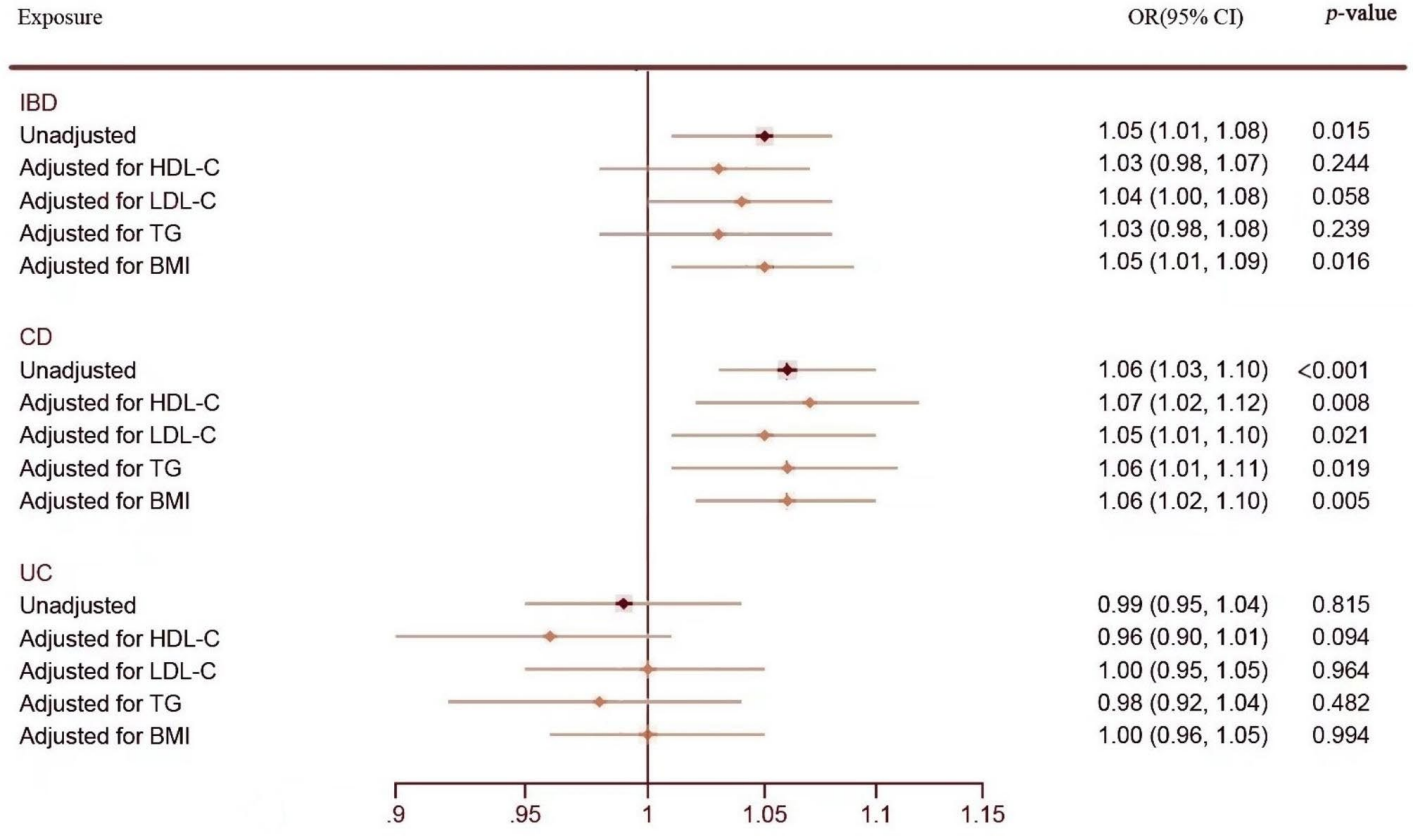
Causal estimates of IBD, CD and UC on KSD in MVMR. Abbreviations: IBD, inflammatory bowel disease; CD, Crohn’s disease; UC, ulcerative colitis; HDL-C: High-density lipoprotein cholesterol; LDL-C: Low-density lipoprotein cholesterol; TG: Triglycerides; BMI: Body mass index; OR, odds ratio

To avoid the influence of pleiotropic SNPs correlated with confounders on causal estimation, we conducted MVMR to adjust for HDL-C, LDL-C, TG and BMI. As with previous findings, MVMR results showed that CD was still associated with an increased risk of KSD after adjusting for each confounding factor, and no direct causal effect of UC on KSD was detected. Although IBD as a whole remained related to an elevated risk of KSD after adjusting for BMI, the positive results turned negative after adjusting for LDL-C, HDL-C and TG (Fig. 5). It suggested that the association between IBD and KSD might be interfered by the serum lipid level in patients. Dyslipidemia might act as a mediator to promote the formation of kidney stones (Fig. 5).

## Discussion

The two-sample bidirectional MR study determined that genetically predicted CD was causally related to KSD, lending support to the findings of epidemiological studies [4, 16, 42, 43]. Whereas our findings indicated that UC was not causally associated with the formation of urinary calculi, somewhat contradicting a few published studies. In the reverse MR analyses, there was no causal evidence supporting the idea that KSD could increase the risk of IBD (including CD and UC). Univariable MR analysis has indicated that IBD as a whole may play a vital role in developing renal calculus. Nevertheless, MVMR analysis refuted the results after adjusting potential confounders including HDL-C, LDL-C and TG, hinting that dyslipidemia might act as a mediator to promote the formation of kidney stones. Relevant studies have shown that patients with IBD have high amounts of inflammatory cytokines in their blood. These inflammatory cytokines may lead to a reduction in lipoprotein lipase enzyme activity, resulting in a typical lipoprotein profile with low levels of HDL-C and elevated levels of LDL-C and TG [44]. However, changes in the patient’s lipid profile may indicate abnormalities in urine physicochemistry and stone risk. For instance, low HDL and high TG levels were linked to reduced urine pH. Non-HDL has a strong relationship with uric acid and urinary sodium. Uric acid stones were more prevalent in patients with high TG levels [32].

Nephrolithiasis is a systemic metabolic disorder, and its main pathogenic factors include metabolic abnormalities, urinary tract infections, and drug factors. IBD (CD and UC) is a kind of idiopathic inflammatory bowel disease characterized by considerable clinical and genetic heterogeneity. Studies have demonstrated the following reasons accounting for the relationship between IBD and KSD: (i) patients with IBD exposed to surgery could lead to supersaturation for calcium oxalate and uric acid [8] and TNF-alpha inhibitor treatment per se drives the increased risk of stone formation [13]; (ii) malabsorption of bile salts and fatty acids caused by recurrent inflammation of the intestine can increase the solubility of oxalate [45]; (iii) chronic intestinal inflammation could lead to fluid losses, bicarbonate losses, and reduced magnesium absorption, which further promote the formation of urinary calculus [46, 47].

According to previous studies, 4–23% of patients with IBD suffered from renal and urinary tract complications, with nephrolithiasis being the most common form of renal manifestation [48]. Based on a meta-analysis including 1624 individuals, urinary complications might occur in up to 22% of patients suffering from IBD, and calcium oxalate is more frequent in patients with CD than with UC [4]. Likewise, the latest retrospective analysis containing 1874 patients diagnosed with IBD reported that renal involvement may be observed in approximately 6% of patients suffering from IBD. Patients with CD seem to be more susceptible than those with UC. It also found renal manifestations were associated with surgical resection history and disease activity in CD patients, whereas no such link was identified in patients with UC [49]. These studies all demonstrated that CD patients presented a higher risk of KSD compared with UC patients. The different effects of CD and UC on KSD might be interpreted as follows: CD belongs to a kind of systemic disorder with a prolonged premorbid stage, whereas UC is frequently confined to the distal colonic tract and characterized by acute mucosal lesions. According to anatomical position, CD occurs primarily in the small intestine and frequently affects the terminal ileum [50], which directly leads to hyperoxaluria in patients with ileal dysfunction [45]. Moreover, extensive resection of the lesions may result in estrogen deficiency [51], which may increase the risk of calculus recurrence by elevating calcium oxalate saturation and urinary calcium [52].

In this MR analysis, the causal impact of UC on KSD was not identified in our outcomes, contradicting most previous studies regarding UC as one of the causes of nephrolithiasis [4, 42]. But in a comparative study, McConnell et al. reported similar findings to ours: renal calculi and hyperoxaluria were found in none of the patients with UC [14]. At the same time, in a recent MR analysis, CD has been shown to sharply increase the risk of urolithiasis, while UC failed to produce this effect [53]. The reasons for the difference between many epidemiological studies and MR analysis are as follows: First, some interference factors may exert an influence on the results of epidemiological observational studies. For instance, the nutritional status and medication status of different groups of people will affect the incidence of kidney stones. Second, MR is an approach using genetic data as a bridge to explore causal associations between exposure and outcome, which is rarely affected by causal inversion and confounding factors. Therefore, it is necessary to implement more epidemiological studies to precisely assess the relationship between UC and KSD. Lastly, publicly available GWAS summary statistics about the effect of disease activity on KSD have not been issued. Thus, it’s difficult to judge whether the MR results were biased by the stage of the disease.

This study had several advantages. Our MR analysis was the first study focusing on this topic. In this research, all the individuals were of European descent, which guaranteed the homology of the population. Meanwhile, two independent populations were utilized to examine these connections, and the consistent findings guaranteed the stability of our results. In addition, various MR methods were used to lend support for exploring the causal impacts of genetically predicted IBD, CD and UC on KSD. Following selection criteria, we screened eligible SNPs as IVs to infer the causal evidence between the risk of interest and outcome after removing LD, outlier SNPs, and pleiotropic genetic variants. To avoid the influence of many weak IVs on our MR analysis, F statistics were calculated and much greater than 10, suggesting a small likelihood of bias caused by weak IVs. With many weak IVs, the MR-RAPS method was also performed to provide a robust inference for the MR results. Since we included two groups of exposure GWAS summary data, the causal estimates might differ from each other, and thus, the combined effect size was obtained using meta-analysis, which augmented the causal inference in terms of IBD (including CD and UC) with the risk of KSD. Lastly, we applied MVMR analyses to adjust confounding factors for investigating the direct causal effect of IBD on KSD and reverse MR to examine the effect of KSD on IBD.

Several limitations existed in this study. First, the exposure and outcome datasets included in the MR analysis referred to data from patients of European descent, which confined the generalizability to other ethnicities. Further investigation is needed to check our conclusions with those of other ancestors. Second, Previous studies have shown minimal renal dysfunction in IBD is related to disease activity but not with 5-ASA use [54], indicating disease activity may play a critical role in extra-intestinal manifestations. In this study, the effect of IBD activity on KSD was unable to be examined with MR analysis due to the lack of GWAS data related to IBD activity. It may not be the disease itself but the IBD activity that is the key promoter of the formation of kidney calculi. Third, our results might be misled on account of the relatively small samples of KSD. Therefore, it must expand to contain data with a larger sample size of nephrolithiasis to study the effect of IBD on KSD in the future. Fourth, although we demonstrated that CD could raise the risk of kidney stone disease, the specific signaling pathways remain in need of more studies. Fifth, overlapping participants are not supposed to be contained in both exposure and outcome datasets applied in two-sample MR analyses. In this research, although the degree of overlap could not be estimated, strong instruments can minimize the bias from population overlap (F statistic>10) [55]. Sixth, it remains uncertain to which degree the risk of KSD is associated with the treatment of IBD or disease severity, and further inclusion in the population for analysis is recommended. Lastly, the important limitation is unobserved pleiotropy, which means the risk of renal calculus might be affected by genetic instruments through other pathways but through IBD, CD and UC, despite the study design being less susceptible to confounders than observational research.

## Conclusion

In summary, this research aimed to evaluate the genetic correlation of IBD (including CD and UC) with KSD using MR analysis. The findings of our study provided genetic evidence in favor of the causal impact of genetically predicted CD on KSD. Particularly, no evidence showed that UC appeared to be associated with an increased risk of nephrolithiasis. The results of the study were of great concern because the clinician’s understanding of the potential risk of developing renal calculus in patients with IBD will facilitate early diagnosis and personalized treatment. Foremost, more advanced approaches to reduce biased estimates and more GWAS summary statistics and RCTs on the disease activity of IBD are warranted to verify our findings in the future.

### Electronic supplementary material

Below is the link to the electronic supplementary material.

**Additional file 1**. Specific characteristics of SNPs screened out for exposure (IBD, CD and UC; IBD (val), CD (val) and UC (val); KSD).

**Additional file 2**. Table S1. Various MR methods and sensitivity analyses for assessing the causal effects of exposure (IBD, CD, UC and KSD) on the outcome (KSD and IBD, CD, UC) and robustness of results. Table S2. Details of SNPs that are significantly correlated with confounding factors (*p*<5E-8). Figure S1. Scatter plots for the causal effect of exposure on KSD. (A) IBD-KSD. (B) CD-KSD. (C) UC-KSD. (D) IBD (val)-KSD. (E) CD (val)-KSD. (F) UC (val)-KSD. Analyses were conducted using the IVW, WM, MR-Egger, MR-RAPS, MR-Egger bootstrap and penalized weighted median methods. The slope of each line represents the MR effect value of the corresponding method. Figure S2. Funnel plots for the causal effect of exposure on KSD. (A) IBD-KSD. (B) CD-KSD. (C) UC-KSD. (D) IBD (val)-KSD. (E) CD (val)-KSD. (F) UC (val)-KSD. Figure S3. “MR-effect” analysis plots for MR analyses of the impact of a single SNP of exposure on KSD. (A) IBD-KSD. (B) IBD (val)-KSD. (C) CD-KSD. (D) CD (val)-KSD. (E) UC-KSD. (F) UC (val)-KSD. Figure S4. “Leave-one-out” analysis plots for the causal impact of exposure on KSD. (A) IBD-KSD. (B) IBD (val)-KSD. (C) CD-KSD. (D) CD (val)-KSD. (E) UC-KSD. (F) UC (val)-KSD. Figure S5. Scatter plots for the causal effect of KSD on the outcome. (A) KSD-IBD. (B) KSD-CD. (C) KSD-UC. Figure S6. Funnel plots for the causal effect of KSD on the outcome. (A) KSD-IBD. (B) KSD-CD. (C) KSD-UC. Figure S7. “MR-effect” analysis plots for MR analyses of the impact of each single SNP of KSD on the outcome. (A) KSD-IBD. (B) KSD-CD. (C) KSD-UC. Figure S8. “Leave-one-out” analysis plots for the causal impact of KSD on the outcome. (A) KSD-IBD. (B) KSD-CD. (C) KSD-UC. Figure S9. Scatter plots of the degree of effect of each SNP on IBD and KSD in MVMR. (A) Adjusting for HDL-C; (B) Adjusting for LDL-C; (C) Adjusting for TG; (D) Adjusting for BMI. Figure S10. Scatter plots of the degree of effect of each SNP on CD and KSD in MVMR. (A) Adjusting for HDL-C; (B) Adjusting for LDL-C; (C) Adjusting for TG; (D) Adjusting for BMI. Figure S11. Scatter plots of the degree of effect of each SNP on UC and KSD in MVMR. (A) Adjusting for HDL-C; (B) Adjusting for LDL-C; (C) Adjusting for TG; (D) Adjusting for BMI.

## Data Availability

All GWAS summary data involved in this study are publicly available in the IEU GWAS databases (https://gwas.mrcieu.ac.uk/): IBD (GWAS ID: ebi-a-GCST004131, ebi-a-GCST003043), CD (GWAS ID: ebi-a-GCST004132, ebi-a-GCST003044), UC (GWAS ID: ebi-a-GCST004133, ebi-a-GCST003045), KSD (GWAS ID: finn-b-N14_CALCUKIDUR), HDL-C (GWAS ID: ieu-b-109), LDL-C (GWAS ID: ieu-b-110), TG (GWAS ID: ieu-b-111), and BMI (GWAS ID: ebi-a-GCST006368).

## Abbreviations

IBD: Inflammatory bowel disease
CD: Crohn’s disease
UC: Ulcerative colitis
KSD: Kidney stone disease
HDL-C: High-density lipoprotein cholesterol
LDL-C: Low-density lipoprotein cholesterol
TG: Triglycerides
BMI: Body mass index
IVW(FE): Inverse variance weighted (fixed effects)
IVW(RE): Inverse variance weighted (random effects)
WM: Weighted median
MR-RAPS: MR Robust Adjusted Profile Score
MR-PRESSO: MR-Pleiotropy RESidual Sum and Outlier method
GWAS: Genome-wide association studies
SNPs: Single-nucleotide polymorphisms
IVs: Instrument variables
LD: Linkage disequilibrium
MR: Mendelian randomization
UVMR: Univariable mendelian randomization
MVMR: Multivariable mendelian randomization
FinnGen: FinnGen consortium
UK Biobank: UK Biobank consortium
IIBDGC: International Inflammatory Bowel Disease Genetics Consortium
RCTs: Randomized controlled trials
val: Validation
SD: Standard deviation
TNF: Tumor necrosis factor
5-ASA: 5-Aminosalicylate
