# Supplementary material for: Causal effects of inflammatory bowel diseases on the risk of kidney stone disease: a two-sample bidirectional mendelian randomization
Source: BMC Urol. 2023 Oct 12;23:162. doi: 10.1186/s12894-023-01332-4 (PMC10571288; doi:10.1186/s12894-023-01332-4)
Supplement: Supplementary file 2 — Additional file 2. Table S1. Various MR methods and sensitivity analyses for assessing the causal effects of exposure (IBD, CD, UC and KSD) on the outcome (KSD and IBD, CD, UC) and robustness of results. Table S2. Details of SNPs that are significantly correlated with confounding factors (p<5E-8). Figure S1. Scatter plots for the causal effect of exposure on KSD. (A) IBD-KSD. (B) CD-KSD. (C) UC-KSD. (D) IBD (val)-KSD. (E) CD (val)-KSD. (F) UC (val)-KSD. Analyses were conducted using the IVW, WM, MR-Egger, MR-RAPS, MR-Egger bootstrap and penalized weighted median methods. The slope of each line represents the MR effect value of the corresponding method. Figure S2. Funnel plots for the causal effect of exposure on KSD. (A) IBD-KSD. (B) CD-KSD. (C) UC-KSD. (D) IBD (val)-KSD. (E) CD (val)-KSD. (F) UC (val)-KSD. Figure S3. “MR-effect” analysis plots for MR analyses of the impact of a single SNP of exposure on KSD. (A) IBD-KSD. (B) IBD (val)-KSD. (C) CD-KSD. (D) CD (val)-KSD. (E) UC-KSD. (F) UC (val)-KSD. Figure S4. “Leave-one-out” analysis plots for the causal impact of exposure on KSD. (A) IBD-KSD. (B) IBD (val)-KSD. (C) CD-KSD. (D) CD (val)-KSD. (E) UC-KSD. (F) UC (val)-KSD. Figure S5. Scatter plots for the causal effect of KSD on the outcome. (A) KSD-IBD. (B) KSD-CD. (C) KSD-UC. Figure S6. Funnel plots for the causal effect of KSD on the outcome. (A) KSD-IBD. (B) KSD-CD. (C) KSD-UC. Figure S7. “MR-effect” analysis plots for MR analyses of the impact of each single SNP of KSD on the outcome. (A) KSD-IBD. (B) KSD-CD. (C) KSD-UC. Figure S8. “Leave-one-out” analysis plots for the causal impact of KSD on the outcome. (A) KSD-IBD. (B) KSD-CD. (C) KSD-UC. Figure S9. Scatter plots of the degree of effect of each SNP on IBD and KSD in MVMR. (A) Adjusting for HDL-C; (B) Adjusting for LDL-C; (C) Adjusting for TG; (D) Adjusting for BMI. Figure S10. Scatter plots of the degree of effect of each SNP on CD and KSD in MVMR. (A) Adjusting for HDL-C; (B) Adjusting for LDL-C; (C) Adjust [file 12894_2023_1332_MOESM2_ESM.docx]

**Table S1.** Various MR methods and sensitivity analyses for assessing the causal effects of exposure (IBD, CD, UC and KSD) on the outcome (KSD and IBD, CD, UC) and results of sensitivity analysis.

**Table S2.** Details of SNPs that are significantly correlated with confounding factors (*p*＜5E-8). **Figure S1.** Scatter plots for the causal effect of exposure on KSD. (A) IBD-KSD. (B) CD-KSD. (C) UC-KSD. (D) IBD (val)-KSD. (E) CD (val)-KSD. (F) UC (val)-KSD. Analyses were conducted using the conventional IVW, WM, MR-Egger, Weighted mode, and Simple mode methods. The slope of each line represents the MR effect value of the corresponding method.

**Figure S2.** Funnel plots for the causal effect of exposure on KSD. (A) IBD-KSD. (B) CD-KSD. (C) UC-KSD. (D) IBD (val)-KSD. (E) CD (val)-KSD. (F) UC (val)-KSD.

**Figure S3.** “MR-effect” analysis plots for MR analyses of the impact of a single SNP of exposure on KSD. (A) IBD-KSD. (B) IBD (val)-KSD. (C) CD-KSD. (D) CD (val)-KSD. (E) UC-KSD. (F) UC (val)-KSD.

**Figure S4.** “Leave-one-out” analysis plots for the causal impact of exposure on KSD. (A) IBD-KSD. (B) IBD (val)-KSD. (C) CD-KSD. (D) CD (val)-KSD. (E) UC-KSD. (F) UC (val)-KSD.

**Figure S5.** Scatter plots for the causal effect of KSD on the outcome. (A) KSD-IBD. (B) KSD-CD. (C) KSD-UC.

**Figure S6.** Funnel plots for the causal effect of KSD on the outcome. (A) KSD-IBD. (B) KSD-CD. (C) KSD-UC.

**Figure S7.** “MR-effect” analysis plots for MR analyses of the impact of each single SNP of KSD on the outcome. (A) KSD-IBD. (B) KSD-CD. (C) KSD-UC.

**Figure S8.** “Leave-one-out” analysis plots for the causal impact of KSD on the outcome. (A) KSD-IBD. (B) KSD-CD. (C) KSD-UC.

**Figure S9.** Scatter plots of the degree of effect of each SNP on IBD and KSD in MVMR. (A) Adjusting for HDL-C; (B) Adjusting for LDL-C; (C) Adjusting for TG; (D) Adjusting for BMI.

**Figure S10.** Scatter plots of the degree of effect of each SNP on CD and KSD in MVMR. (A) Adjusting for HDL-C; (B) Adjusting for LDL-C; (C) Adjusting for TG; (D) Adjusting for BMI. **Figure S11.** Scatter plots of the degree of effect of each SNP on UC and KSD in MVMR. (A) Adjusting for HDL-C; (B) Adjusting for LDL-C; (C) Adjusting for TG; (D) Adjusting for BMI.

**Table S1.** Various MR methods and sensitivity analyses for assessing the causal effects of exposure (IBD, CD, UC and KSD) on outcome (KSD and IBD, CD, UC) and robustness of results.

| **Exposure** | **Outcome** | **nSNPs** | **Method** | **OR (95%CI)** | ***p*** | **Heterogeneity** | | | **Pleiotropy test** | **MR-Presso** | **F(min-max)** |
| --- | --- | --- | --- | --- | --- | --- | --- | --- | --- | --- | --- |
|  |  |  |  |  |  | **Method** | **Q(df)** | ***p*** | ***p*-value** | ***p*-value** |  |
| IBD | KSD | 98 | MR-Egger | 1.05(0.99-1.12) | 0.108 | MR-Egger | 104.0(96) | 0.271 | 0.812 | 0.306 | 46.1(29.9-500.6) |
|  |  |  | MR-Egger (bootstrap) | 1.00(0.93-1.08) | 0.477 | IVW | 104.1(97) | 0.294 |  |  |  |
|  |  |  | WM | 1.07(1.00-1.14) | 0.039 |  |  |  |  |  |  |
|  |  |  | Penalized WM | 1.07(1.00-1.14) | 0.040 |  |  |  |  |  |  |
|  |  |  | IVW-FE | 1.05(1.01-1.08) | 0.012 |  |  |  |  |  |  |
|  |  | 94 | MR-RAPS  IVW-FE* | 1.05(1.01-1.08)  1.05(1.01-1.09) | 0.020  0.010 |  |  |  |  |  |  |
| CD | KSD | 75 | MR-Egger | 1.00(0.90-1.11) | 0.978 | MR-Egger | 84.9(73) | 0.161 | 0.214 | 0.148 | 55.4(30.1-489.6) |
|  |  |  | MR-Egger (bootstrap) | 1.00(0.92-1.09) | 0.470 | IVW | 86.8(74) | 0.147 |  |  |  |
|  |  |  | WM | 1.03(0.98-1.10) | 0.243 |  |  |  |  |  |  |
|  |  |  | Penalized WM | 1.03(0.98-1.09) | 0.239 |  |  |  |  |  |  |
|  |  |  | IVW-FE | 1.06(1.03-1.10) | <0.001 |  |  |  |  |  |  |
|  |  | 69 | MR-RAPS  IVW-FE* | 1.06(1.02-1.10)  1.07(1.03-1.11) | 0.005  < 0.001 |  |  |  |  |  |  |
| UC | KSD | 48 | MR-Egger | 0.96(0.84-1.10) | 0.575 | MR-Egger | 35.1(46) | 0.880 | 0.609 | 0.892 | 59.3(30.5-408.1) |
|  |  |  | MR-Egger (bootstrap) | 0.98(0.87-1.10) | 0.361 | IVW | 35.3(47) | 0.894 |  |  |  |
|  |  |  | WM | 0.98(0.91-1.04) | 0.474 |  |  |  |  |  |  |

**Table S1.** Various MR methods and sensitivity analyses for assessing the causal effects of exposure (IBD, CD, UC and KSD) on outcome (KSD and IBD, CD, UC) and robustness of results. (Continued)

| **Exposure** | **Outcome** | **nSNPs** | **Method** | **OR (95%CI)** | ***p*** | **Heterogeneity** | | | **Pleiotropy test** | **MR-Presso** | **F(min-max)** |
| --- | --- | --- | --- | --- | --- | --- | --- | --- | --- | --- | --- |
|  |  |  |  |  |  | **Method** | **Q(df)** | ***p*** | ***p*-value** | ***p*-value** |  |
|  |  |  | Penalized WM | 0.98(0.91-1.05) | 0.493 |  |  |  |  |  |  |
|  |  |  | IVW-FE | 0.99(0.95-1.04) | 0.815 |  |  |  |  |  |  |
|  |  |  | MR-RAPS | 0.99(0.95-1.04) | 0.682 |  |  |  |  |  |  |
| IBD (val) | KSD | 123 | MR-Egger | 0.96(0.87-1.07) | 0.477 | MR-Egger | 140.2(121) | 0.112 | 0.151 | 0.101 | 57.2(30.3-774.8) |
|  |  |  | MR-Egger (bootstrap) | 0.96(0.87-1.07) | 0.232 | IVW | 142.6(122) | 0.098 |  |  |  |
|  |  |  | WM | 1.01(0.94-1.07) | 0.869 |  |  |  |  |  |  |
|  |  |  | Penalized WM | 1.00(0.94-1.07) | 0.898 |  |  |  |  |  |  |
|  |  |  | IVW-FE | 1.03(0.99-1.07) | 0.120 |  |  |  |  |  |  |
|  |  | 116 | MR-RAPS  IVW-FE* | 1.03(0.99-1.07)  1.03(0.98-1.07) | 0.221  0.231 |  |  |  |  |  |  |
| CD (val) | KSD | 109 | MR-Egger | 1.02(0.91-1.13) | 0.743 | MR-Egger | 131.8(107) | 0.052 | 0.593 | 0.069 | 61.5(30.7-724.5) |
|  |  |  | MR-Egger (bootstrap) | 1.01(0.94-1.09) | 0.375 | IVW | 132.1(108) | 0.057 |  |  |  |
|  |  |  | WM | 1.02(0.97-1.09) | 0.410 |  |  |  |  |  |  |
|  |  |  | Penalized WM | 1.02(0.96-1.09) | 0.477 |  |  |  |  |  |  |
|  |  |  | IVW-FE | 1.05(1.01-1.08) | 0.013 |  |  |  |  |  |  |
|  |  | 102 | MR-RAPS  IVW-FE* | 1.04(1.00-1.09)  1.04(1.01-1.08) | 0.068  0.019 |  |  |  |  |  |  |

**Table S1.** Various MR methods and sensitivity analyses for assessing causal effects of exposure (IBD, CD, UC and KSD) on outcome (KSD and IBD, CD, UC) and robustness of results. (Continued)

| **Exposure** | **Outcome** | **nSNPs** | **Method** | **OR (95%CI)** | ***p*** | **Heterogeneity** | | | **Pleiotropy test** | **MR-Presso** | **F(min-max)** |
| --- | --- | --- | --- | --- | --- | --- | --- | --- | --- | --- | --- |
|  |  |  |  |  |  | **Method** | **Q(df)** | ***p*** | ***p*-value** | ***p*-value** |  |
| UC (val) | KSD | 82 | MR-Egger | 1.03(0.92-1.15) | 0.637 | MR-Egger | 90.5(80) | 0.198 | 0.486 | 0.220 | 52.1(29.8-425.8) |
|  |  |  | MR-Egger (bootstrap) | 1.02(0.93-1.12) | 0.301 | IVW | 91.0(81) | 0.209 |  |  |  |
|  |  |  | WM | 1.00(0.94-1.07) | 0.982 |  |  |  |  |  |  |
|  |  |  | Penalized WM | 1.00(0.94-1.07) | 0.984 |  |  |  |  |  |  |
|  |  |  | IVW-FE | 0.99(0.95-1.03) | 0.665 |  |  |  |  |  |  |
|  |  | 80 | MR-RAPS  IVW-FE* | 0.99(0.94-1.04)  0.99(0.95-1.04) | 0.726  0.784 |  |  |  |  |  |  |
| KSD | IBD | 26 | MR-Egger | 1.09(1,00-1.19) | 0.058 | MR-Egger | 33.4(24) | 0.096 | 0.170 | 0.061 | 22.1(20.9-38.6) |
|  |  |  | MR-Egger (bootstrap) | 0.99(0.89-1.11) | 0.450 | IVW | 36.2(25) | 0.069 |  |  |  |
|  |  |  | WM | 1.05(0.98-1.12) | 0.170 |  |  |  |  |  |  |
|  |  |  | Penalized WM | 1.05(0.98-1.12) | 0.164 |  |  |  |  |  |  |
|  |  |  | IVW-FE | 1.04(1.00-1.08) | 0.082 |  |  |  |  |  |  |
|  |  |  | MR-RAPS | 1.04(0.99-1.09) | 0.137 |  |  |  |  |  |  |
| KSD | CD | 26 | MR-Egger | 1.10(0.99-1.21) | 0.085 | MR-Egger | 28.0(24) | 0.261 | 0.166 | 0.170 | 22.1(20.9-38.6) |
|  |  |  | MR-Egger (bootstrap) | 0.96(0.83-1.11) | 0.308 | IVW | 30.3(25) | 0.212 |  |  |  |
|  |  |  | WM | 1.04(0.95-1.13) | 0.450 |  |  |  |  |  |  |
|  |  |  | Penalized WM | 1.04(0.96-1.13) | 0.351 |  |  |  |  |  |  |

**Table S1.** Various MR methods and sensitivity analyses for assessing the causal effects of exposure (IBD, CD, UC and KSD) on outcome (KSD and IBD, CD, UC) and robustness of results. (Continued)

| **Exposure** | **Outcome** | **nSNPs** | **Method** | **OR (95%CI)** | ***p*** | **Heterogeneity** | | | **Pleiotropy test** | **MR-Presso** | **F(min-max)** |
| --- | --- | --- | --- | --- | --- | --- | --- | --- | --- | --- | --- |
|  |  |  |  |  |  | **Method** | **Q(df)** | ***p*** | ***p*-value** | ***p*-value** |  |
|  |  |  | IVW-FE | 1.03(0.98-1.09) | 0.234 |  |  |  |  |  |  |
|  |  |  | MR-RAPS | 1.03(0.96-1.09) | 0.412 |  |  |  |  |  |  |
| KSD | UC | 24 | MR-Egger | 1.09(0.97-1.23) | 0.178 | MR-Egger | 35.5(22) | 0.034 | 0.481 | 0.047 | 22.1(20.9-38.6) |
|  |  |  | MR-Egger (bootstrap) | 1.06(0.92-1.23) | 0.224 | IVW | 36.3(23) | 0.038 |  |  |  |
|  |  |  | WM | 1.11(1,02-1.20) | 0.013 |  |  |  |  |  |  |
|  |  |  | Penalized WM | 1.11(1,02-1.20) | 0.011 |  |  |  |  |  |  |
|  |  |  | IVW-RE | 1.05(0.98-1.12) | 0.157 |  |  |  |  |  |  |
|  |  |  | MR-RAPS | 1.06(1.00-1.14) | 0.068 |  |  |  |  |  |  |

OR, odds ratio; nSNPs, number of single-nucleotide polymorphisms; CI, confidence interval; MR-Presso, MR-Pleiotropy Sum and Outlier method; MR-RAPS, MR-Robust Adjusted Profile Score; IVW-FE, inverse variance weighted with fixed effects; IVW-RE, inverse variance weighted with random-effects effects; val, validation; IBD, inflammatory bowel disease; CD, Crohn’s disease; UC, ulcerative colitis; KSD, kidney stone disease; F(min-max), the median of F statistics (minimum, maximum), F = β2 exposure / SE2 exposure; Recalculation of causality by IVW method after excluding pleiotropic SNPs identified in PhenoScanner; ^#^ No pleiotropic SNPs were detected

**Table S2.** Details of SNPs that are significantly correlated with confounding factors (*p*＜5E-8).

| **Exposure** | **SNP** | **EA/NEA** | **Trait** | **Beta** | ***P*-value** |
| --- | --- | --- | --- | --- | --- |
| IBD | rs11066188 | A/G | Total cholesterol | -0.0293 | 9.89E-15 |
|  |  |  | Low-density lipoprotein | -0.0258 | 7.99E-11 |
|  |  |  | LDL cholesterol | NA | 2.15E-09 |
|  |  |  | High-density lipoprotein | -0.0209 | 1.20E-08 |
|  | rs28374519 | A/G | Body mass index | -0.01782 | 7.87E-13 |
|  | rs5754100 | C/T | HDL cholesterol | NA | 2.56E-08 |
|  |  |  | High-density lipoprotein | -0.0342 | 2.56E-08 |
|  | rs76286777 | C/T | Body mass index | 0.03278 | 1.53E-29 |
| CD | rs13107325 | T/C | Body mass index | 0.055 | 2.80E-35 |
|  |  |  | HDL cholesterol | 0.071 | 1.00E-15 |
|  |  |  | High-density lipoprotein | -0.0708 | 1.06E-15 |
|  | rs1583792 | C/T | Body mass index | -0.01393 | 7.40E-09 |
|  | rs42861 | G/A | Body mass index | 0.02406 | 2.43E-23 |
|  | rs4343432 | A/G | Body mass index | -0.03495 | 7.12E-48 |
|  | rs492602 | G/A | Total cholesterol | 0.031 | 1.00E-16 |
|  |  |  | Low-density lipoprotein | 0.0293 | 9.42E-14 |
|  |  |  | LDL cholesterol | NA | 4.69E-08 |
|  | rs5754100 | C/T | High-density lipoprotein | -0.0342 | 2.56E-08 |
|  |  |  | HDL cholesterol | NA | 2.56E-08 |
| UC | - |  | - | - | - |
| IBD (val) | rs13407913 | A/G | Body mass index | -0.03176 | 1.60E-39 |
|  | rs3184504 | C/T | Total cholesterol | 0.0318 | 1.62E-17 |
|  |  |  | High-density lipoprotein | 0.0258 | 4.10E-12 |
|  |  |  | Low-density lipoprotein | 0.0268 | 4.20E-12 |
|  |  |  | LDL cholesterol | NA | 1.30E-11 |
|  |  |  | Body mass index | 0.013 | 1.60E-09 |
|  | rs4795397 | A/G | High-density lipoprotein | -0.0222 | 1.03E-09 |
|  | rs516246 | T/C | Total cholesterol | 0.0315 | 9.13E-17 |
|  | rs62037363 | C/T | Body mass index | 0.0264 | 4.21E-27 |
|  | rs780094 | T/C | Triglycerides | 0.1102 | 2.65E-220 |
|  |  |  | Total cholesterol | 0.0504 | 5.28E-41 |
|  | rs9273363 | A/C | Body mass index | -0.02679 | 1.08E-13 |
|  |  |  | Total cholesterol | -0.0232 | 1.65E-08 |
|  |  |  | Low-density lipoprotein | -0.0244 | 1.80E-08 |
| CD (val) | rs13407913 | A/G | Body mass index | -0.03176 | 1.60E-39 |
|  | rs17391694 | C/T | Body mass index | -0.034 | 6.70E-26 |
|  | rs26528 | T/C | Body mass index | -0.0224 | 1.42E-20 |
|  | rs3184504 | C/T | Total cholesterol | 0.0318 | 1.62E-17 |
|  |  |  | High-density lipoprotein | 0.0258 | 4.10E-12 |
|  |  |  | Low-density lipoprotein | 0.0268 | 4.20E-12 |
|  |  |  | LDL cholesterol | NA | 1.30E-11 |
|  |  |  | Body mass index | 0.013 | 1.60E-09 |
|  | rs4795397 | A/G | High-density lipoprotein | -0.0222 | 1.03E-09 |
|  | rs516246 | T/C | Total cholesterol | 0.0315 | 9.13E-17 |
|  |  |  | Low-density lipoprotein | 0.0291 | 1.33E-13 |
|  | rs780094 | T/C | Triglycerides | 0.1102 | 2.65E-220 |
|  |  |  | Total cholesterol | 0.0504 | 5.28E-41 |
| UC (val) | rs2516440 | G/A | Total cholesterol | -0.028 | 2.54E-12 |
|  | rs4795397 | A/G | High-density lipoprotein | -0.0222 | 1.03E-09 |

SNPs, single-nucleotide polymorphisms; IBD, inflammatory bowel disease; CD, Crohn’s disease; UC, ulcerative colitis; val, validation; HDL, high-density lipoprotein; LDL, low-density lipoprotein

**Figure S1.** Scatter plots for the causal effect of exposure on KSD. (A) IBD-KSD. (B) CD-KSD. (C) UC-KSD. (D) IBD (val)-KSD. (E) CD (val)-KSD. (F) UC (val)-KSD.


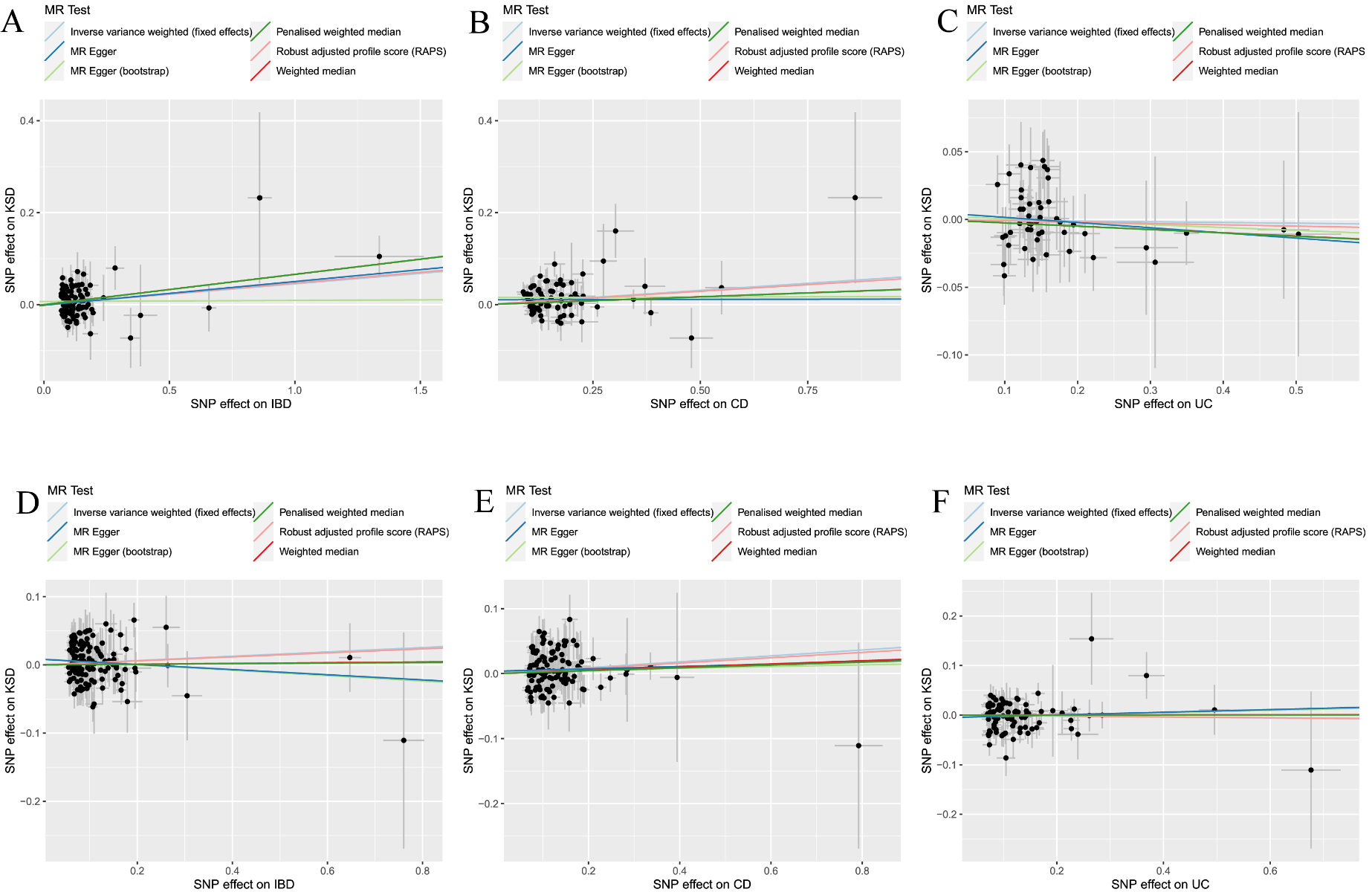


MR, mendelian randomization; IBD, inflammatory bowel disease; CD, Crohn’s disease; UC, ulcerative colitis; val, validation; KSD, kidney stone disease

**Figure S2.** Funnel plots for the causal effect of exposure on KSD. (A) IBD-KSD. (B) CD-KSD. (C) UC-KSD. (D) IBD (val)-KSD. (E) CD (val)-KSD. (F) UC (val)-KSD.

**
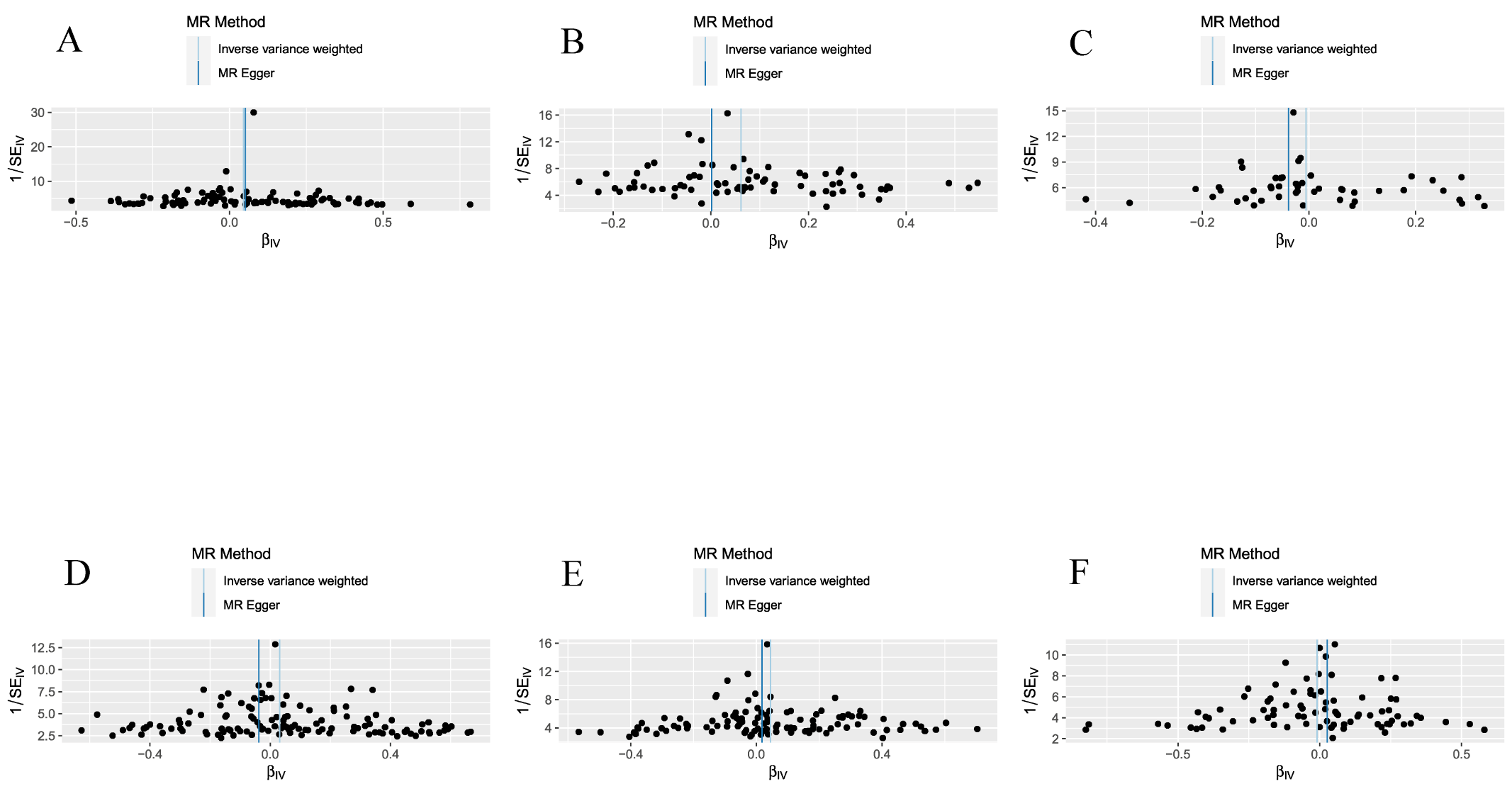
**

MR, mendelian randomization; IBD, inflammatory bowel disease; CD, Crohn’s disease; UC, ulcerative colitis; val, validation; KSD, kidney stone disease

**Figure S3.** “MR-effect” analysis plots for MR analyses of the impact of each single SNP of exposure on KSD. (A) IBD-KSD. (B) IBD (val)-KSD.

**
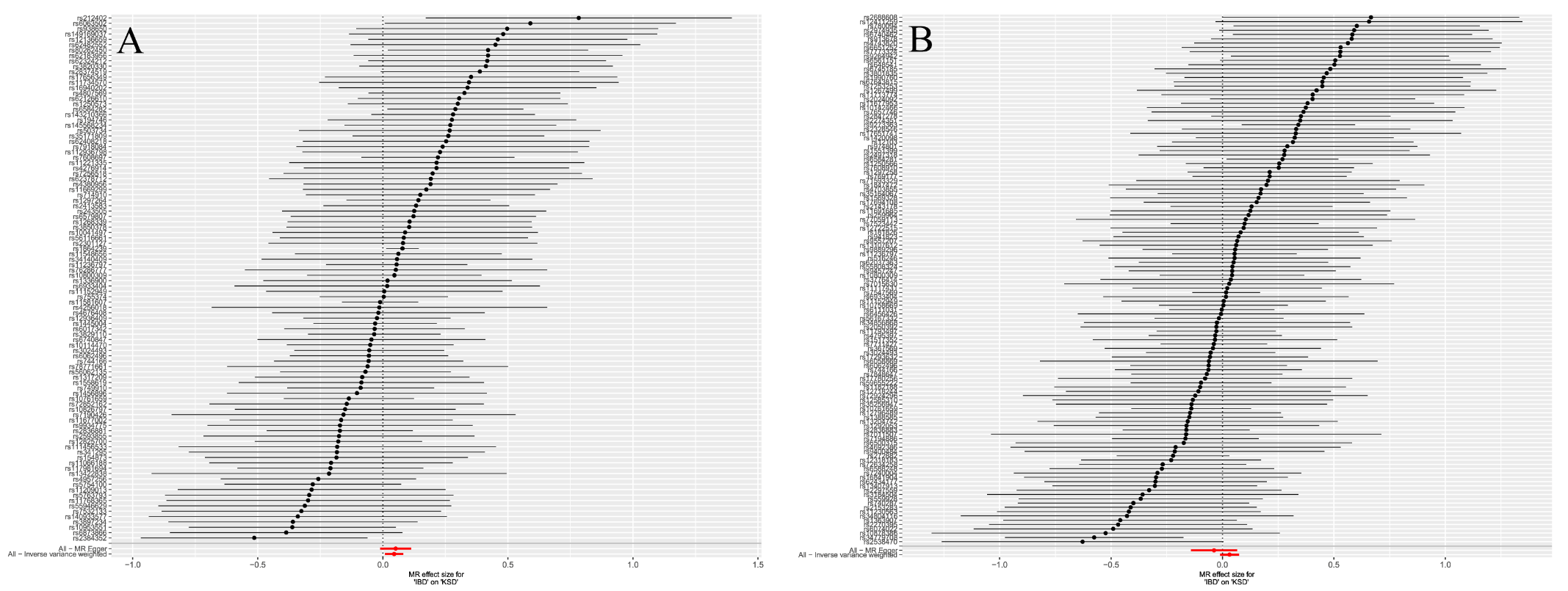
**

MR, mendelian randomization; SNP, single-nucleotide polymorphism; IBD, inflammatory bowel disease; val, validation; KSD, kidney stone disease

**Figure S3.** “MR-effect” analysis plots for MR analyses of the impact of a single SNP of exposure on KSD. (Continued) (C) CD-KSD. (D) CD (val)-KSD.
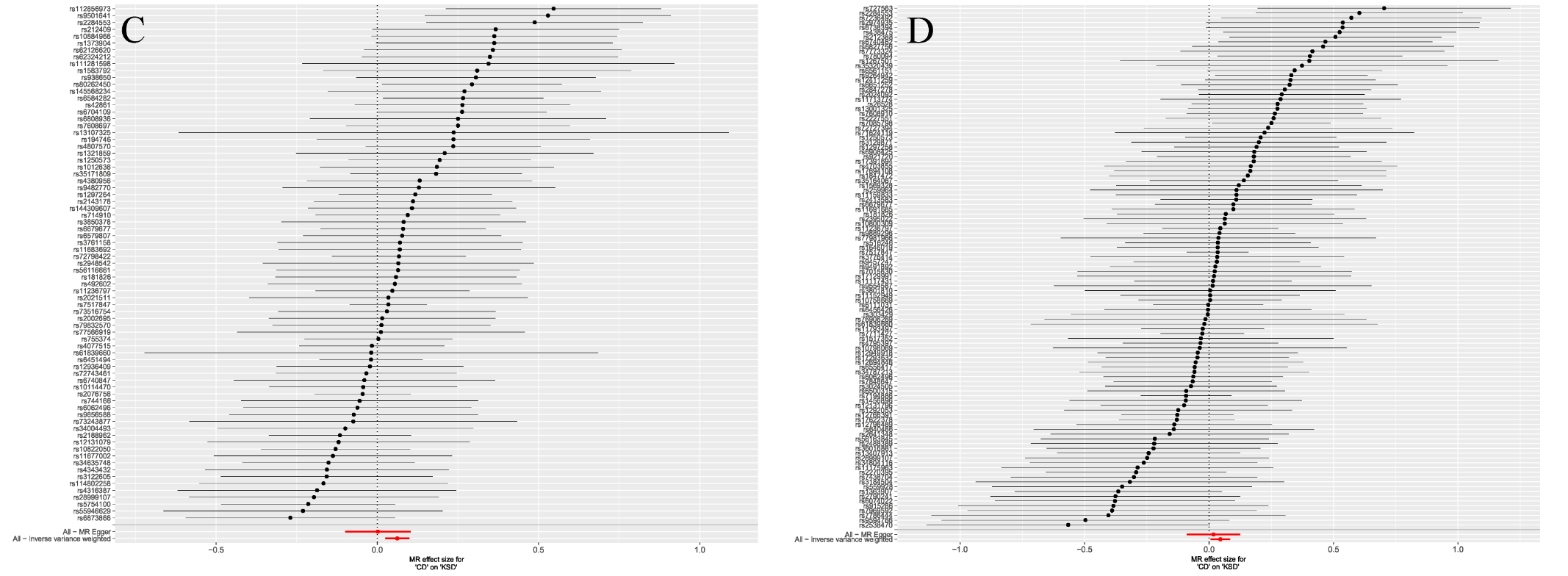
 MR, mendelian randomization; SNP, single-nucleotide polymorphism; CD, Crohn’s disease; val, validation; KSD, kidney stone disease

**Figure S3.** “MR-effect” analysis plots for MR analyses of the impact of a single SNP of exposure on KSD. (Continued) (E) UC-KSD. (F) UC (val)-KSD.


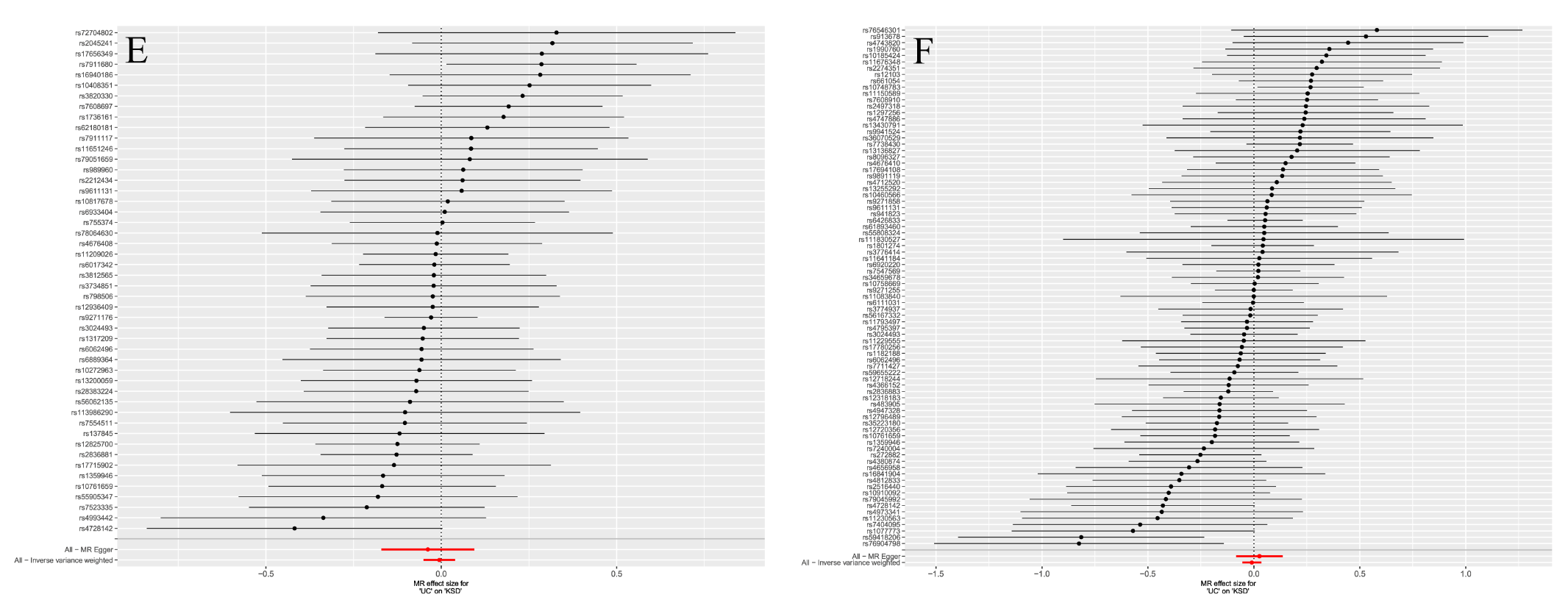


MR, mendelian randomization; SNP, single-nucleotide polymorphism;UC, ulcerative colitis; val, validation; KSD, kidney stone disease

**Figure S4.** “Leave-one-out” analysis plots for the causal impact of exposure on KSD. (A) IBD-KSD. (B) IBD (val)-KSD.

**
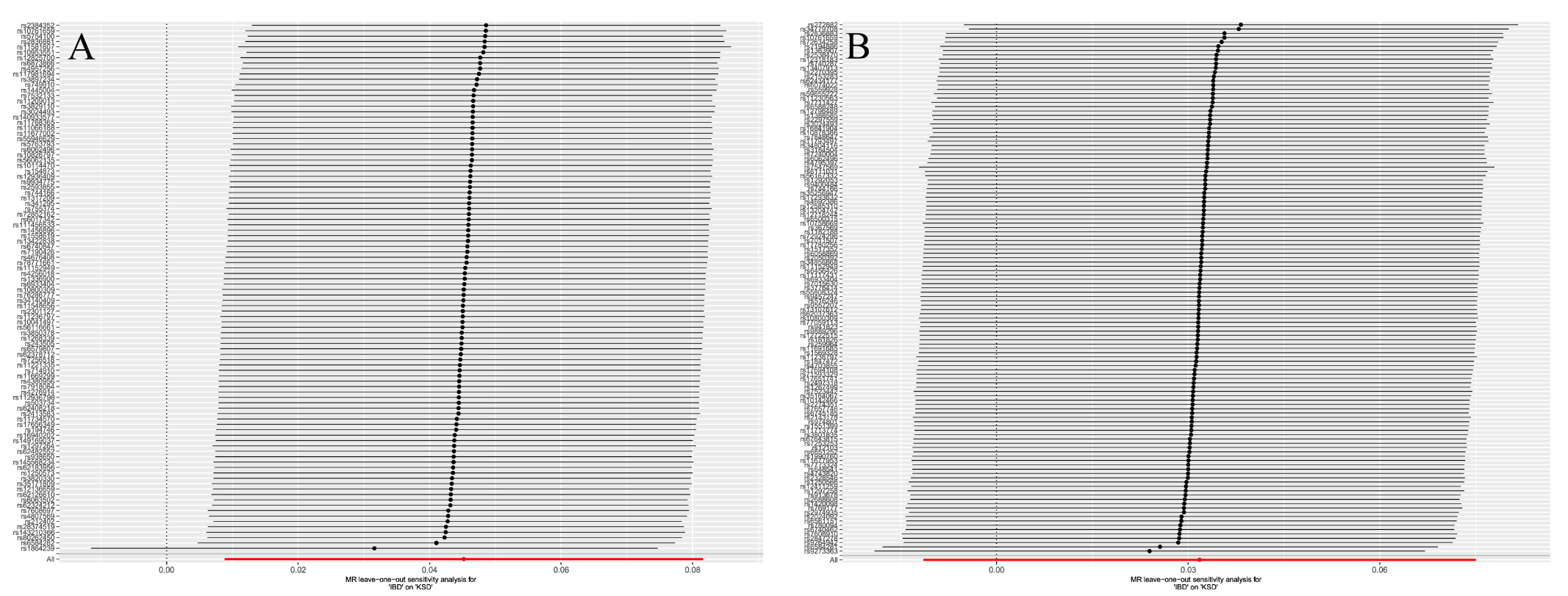
**

MR, mendelian randomization; IBD, inflammatory bowel disease; val, validation; KSD, kidney stone disease

**Figure S4.** “Leave-one-out” analysis plots for the causal impact of exposure on KSD. (Continued) (C) CD-KSD. (D) CD (val)-KSD.


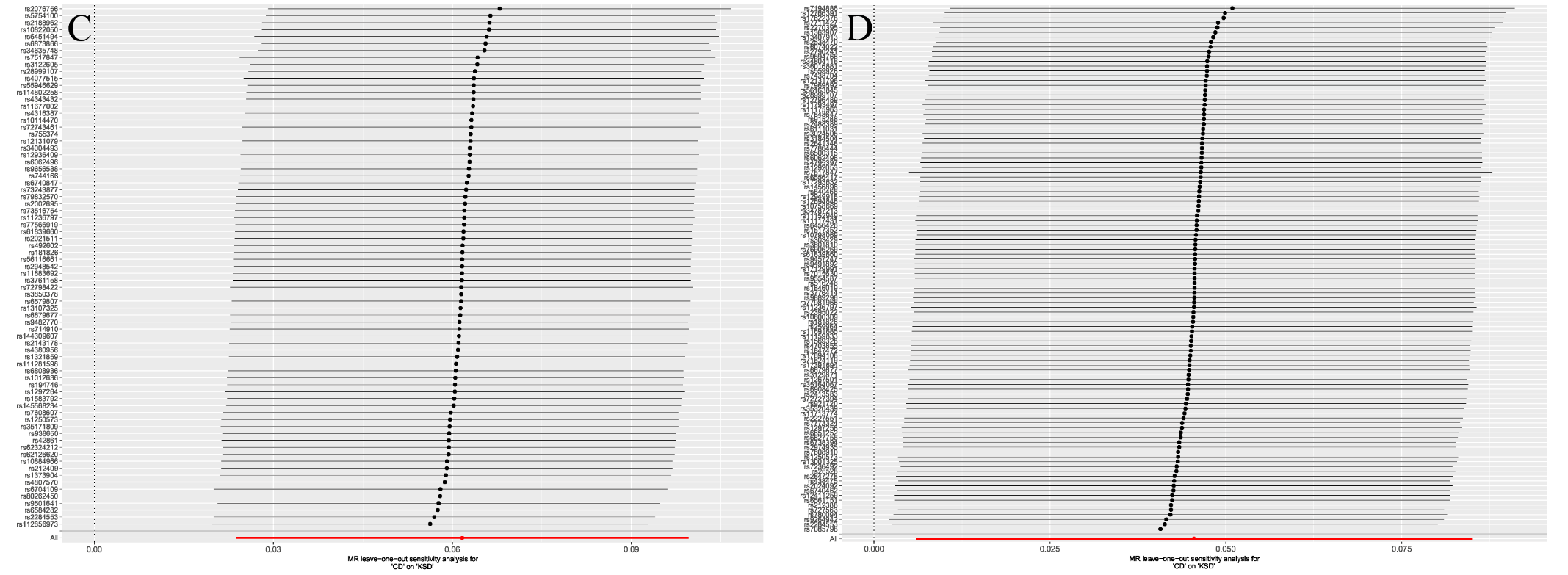
MR, mendelian randomization; CD, Crohn’s disease; val, validation; KSD, kidney stone disease

**Figure S4.** “Leave-one-out” analysis plots for the causal impact of exposure on KSD. (Continued) (E) UC-KSD. (F) UC (val)-KSD.


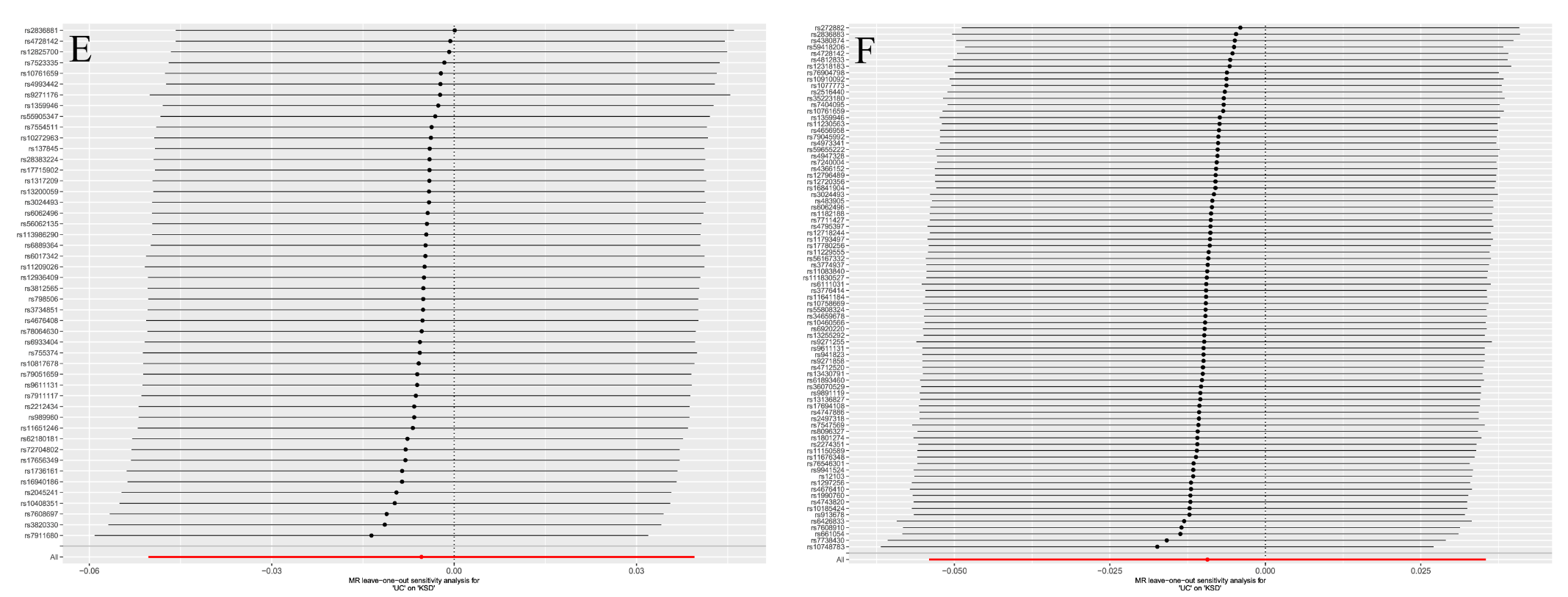
MR, mendelian randomization; UC, ulcerative colitis; val, validation; KSD, kidney stone disease

**Figure S5.** Scatter plots for the causal effect of KSD on the outcome. (A) KSD-IBD. (B) KSD-CD. (C) KSD-UC.


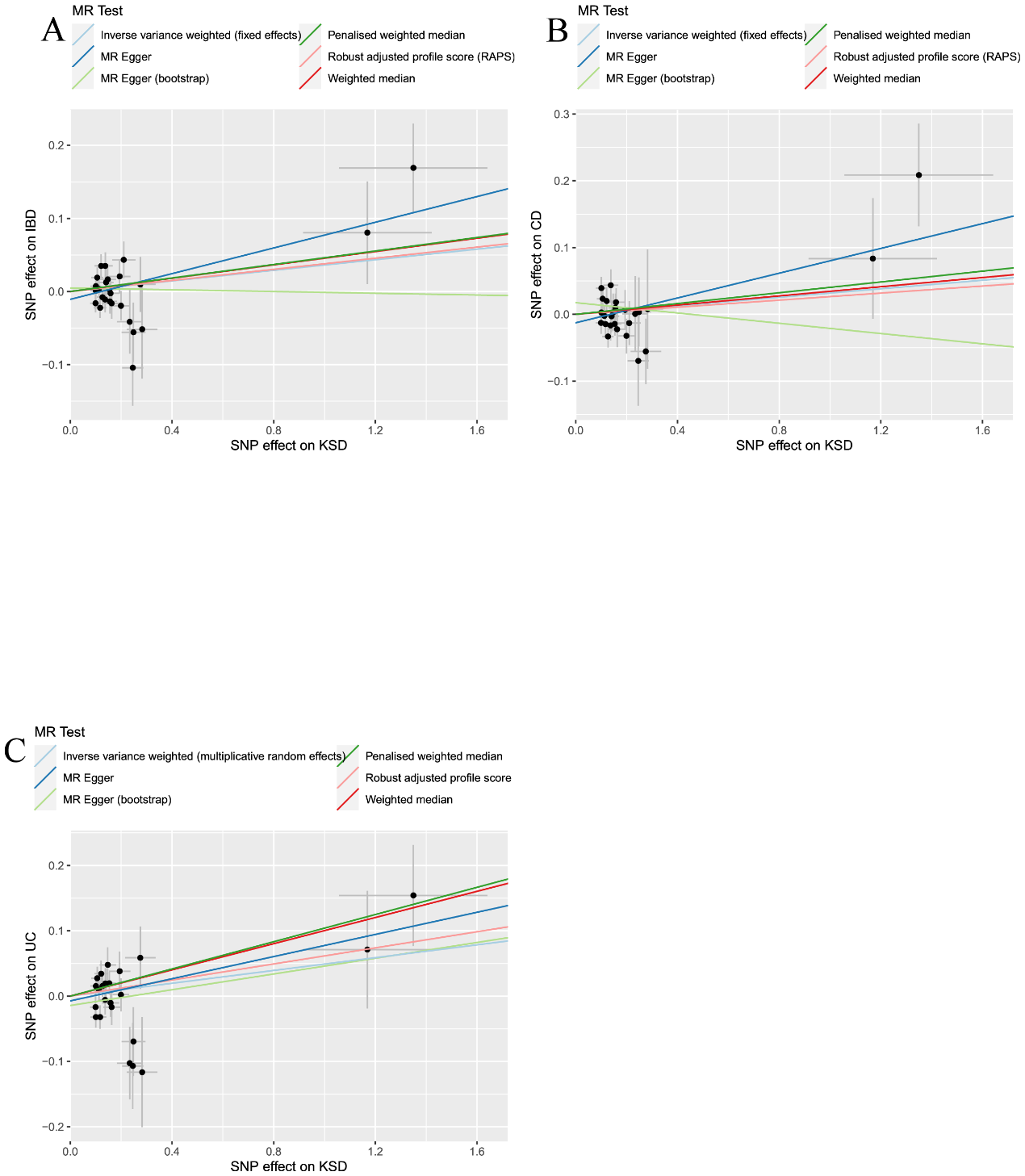


**Figure S6.** Funnel plots for the causal effect of KSD on the outcome. (A) KSD-IBD. (B) KSD-CD. (C) KSD-UC.


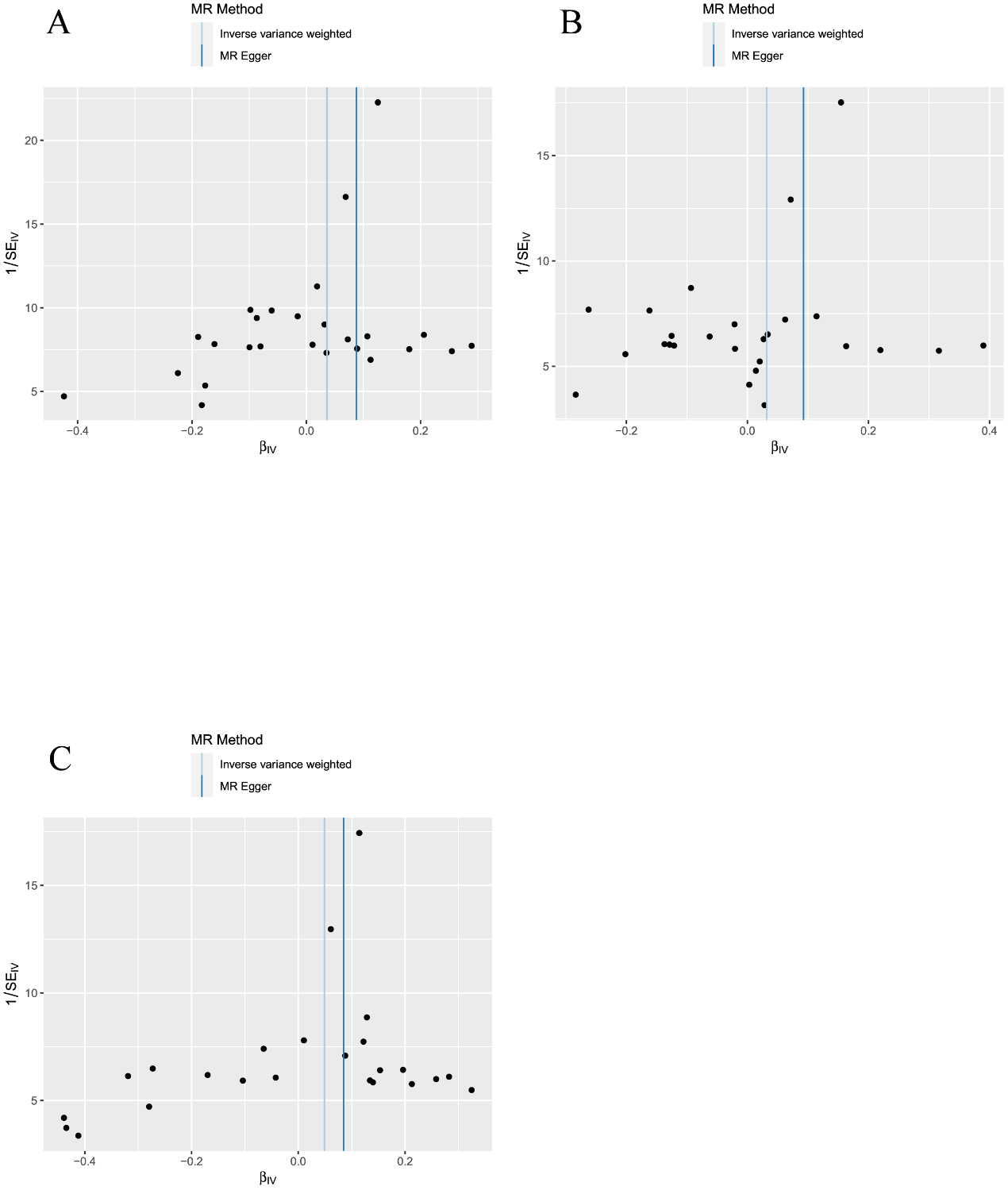


**Figure S7.** “MR-effect” analysis plots for MR analyses of the impact of each single SNP of KSD on the outcome. (A) KSD-IBD. (B) KSD-CD. (C) KSD-UC.

**
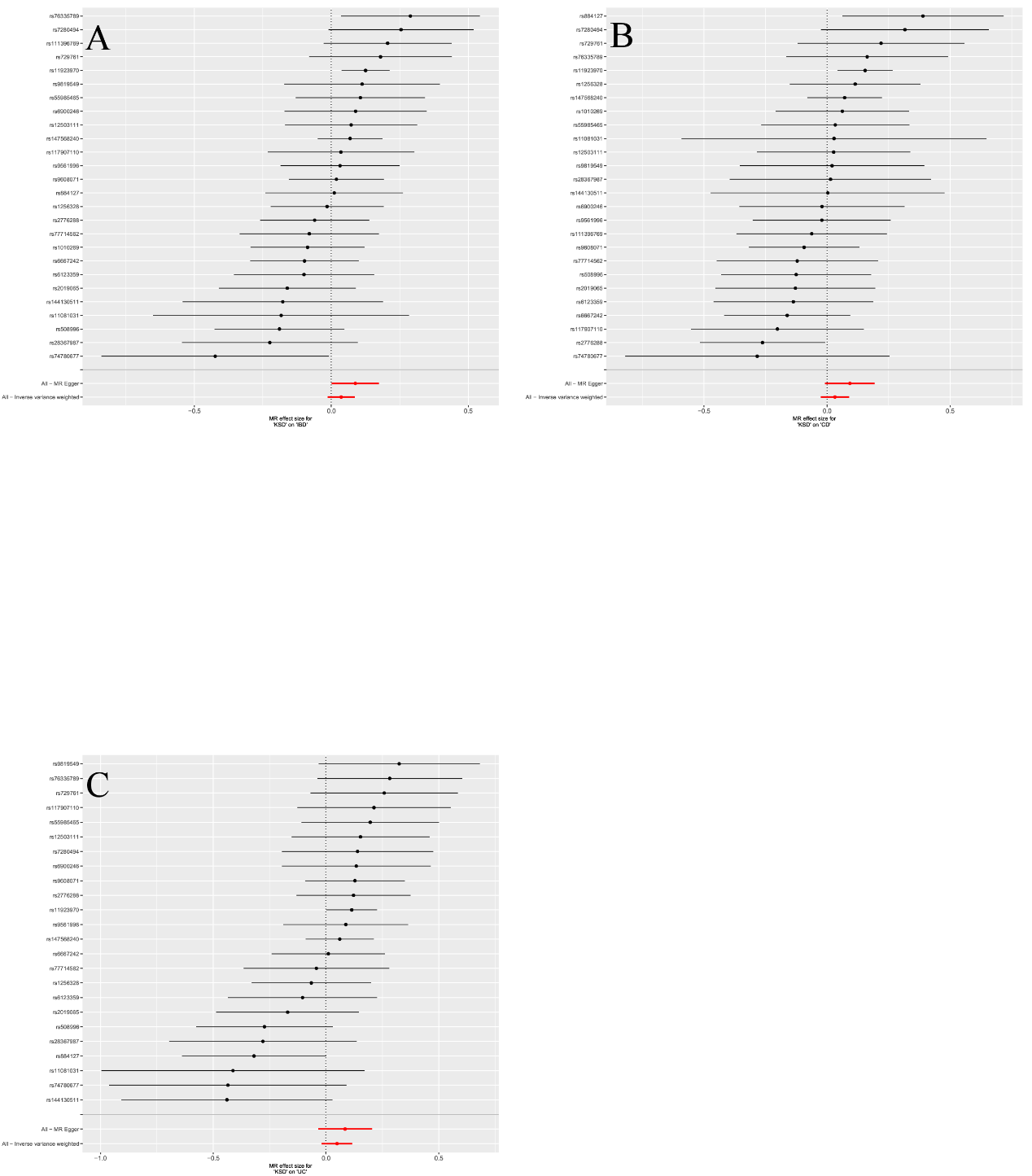
**

**Figure S8.** “Leave-one-out” analysis plots for the causal impact of KSD on the outcome. (A) KSD-IBD. (B) KSD-CD. (C) KSD-UC.

**
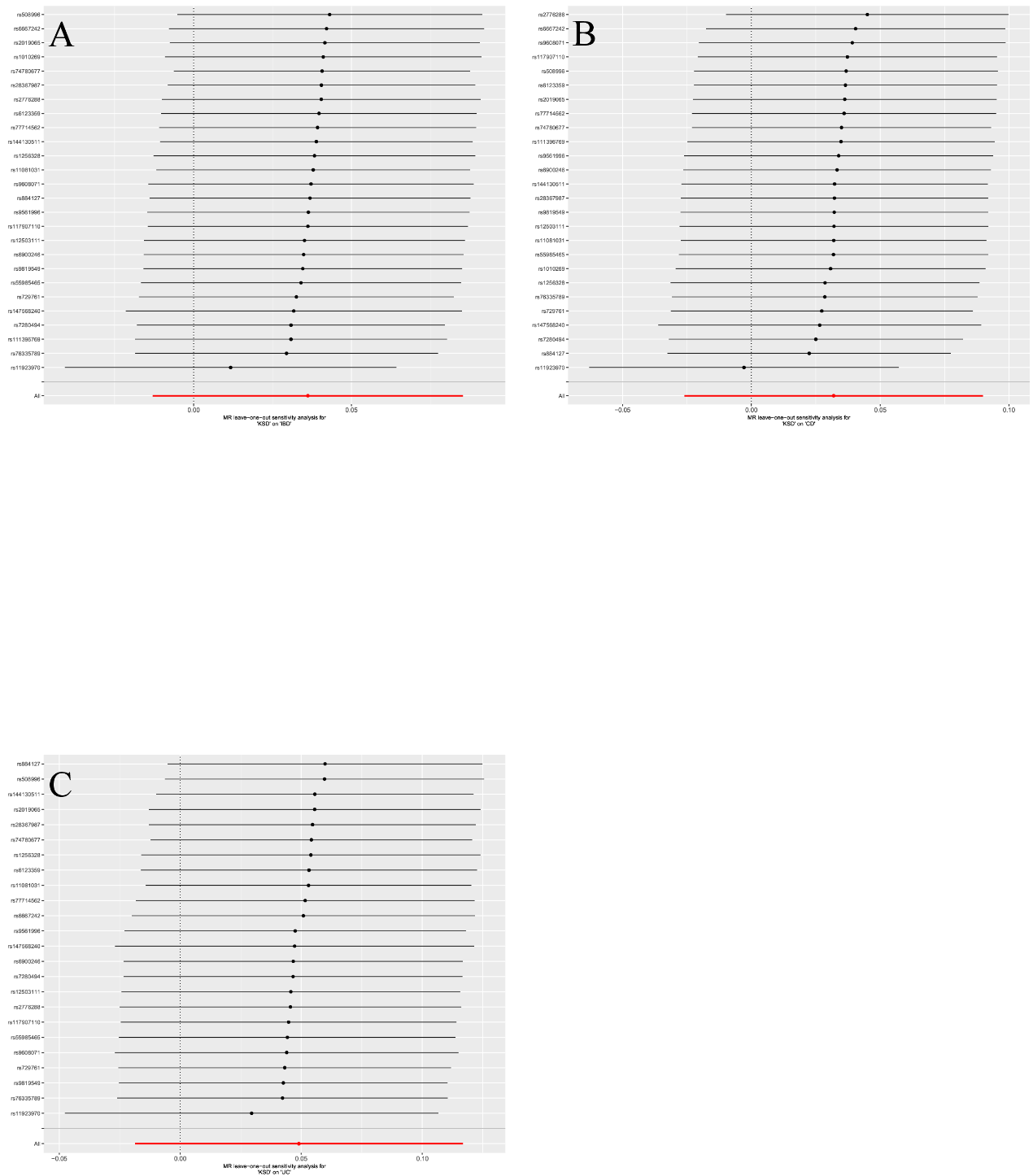
**

**Figure S9.** Scatter plots of the degree of effect of each SNP on IBD and KSD in MVMR. (A) Adjusting for HDL-C; (B) Adjusting for LDL-C; (C) Adjusting for TG; (D) Adjusting for BMI.


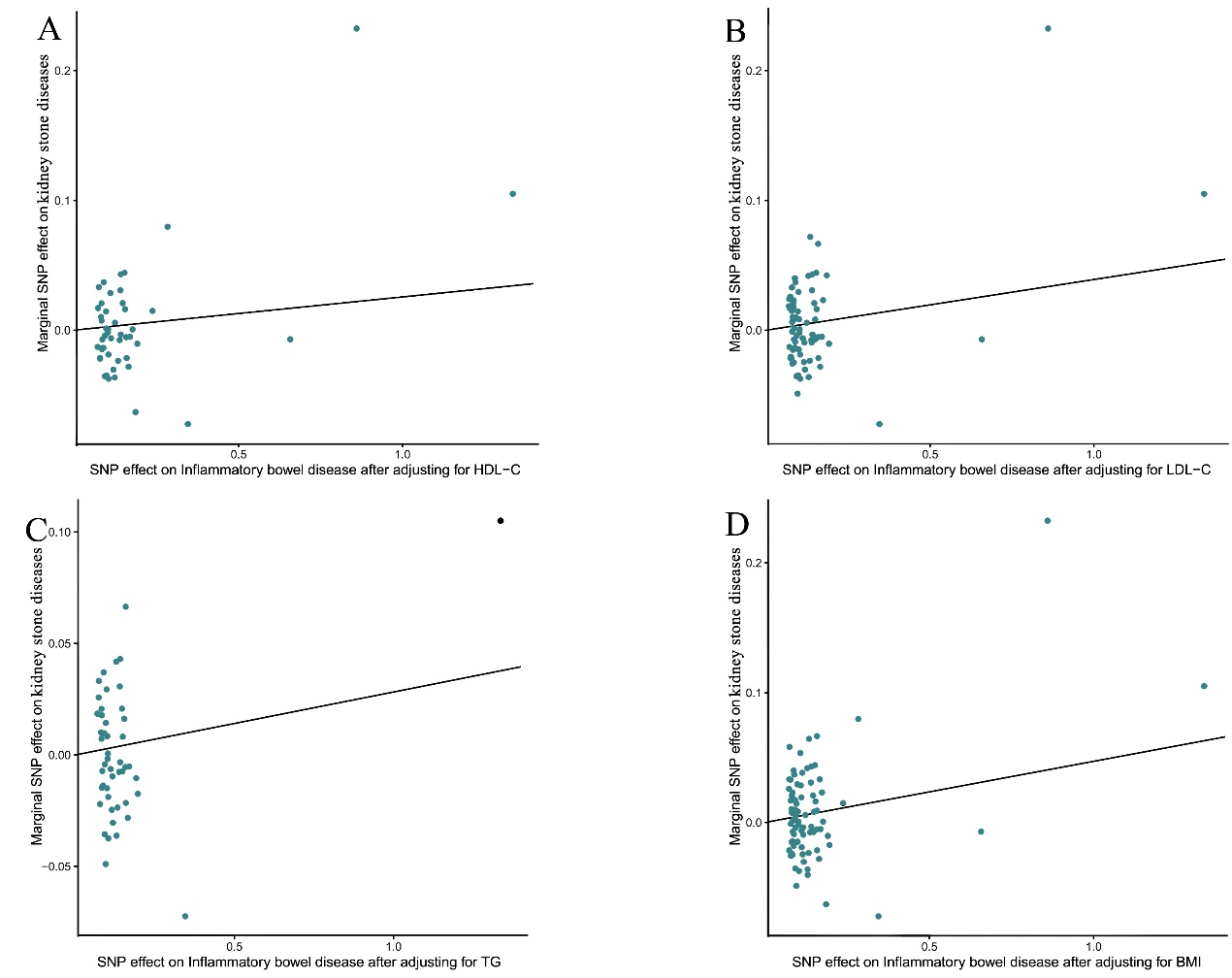


MVMR, multivariable mendelian randomization; SNP, single-nucleotide polymorphism; IBD, inflammatory bowel disease; KSD, kidney stone disease; HDL-C, high-density lipoprotein cholesterol; LDL-C, low-density lipoprotein cholesterol; TG, triglycerides; BMI, body mass index

**Figure S10.** Scatter plots of the degree of effect of each SNP on CD and KSD in MVMR. (A) Adjusting for HDL-C; (B) Adjusting for LDL-C; (C) Adjusting for TG; (D) Adjusting for BMI.


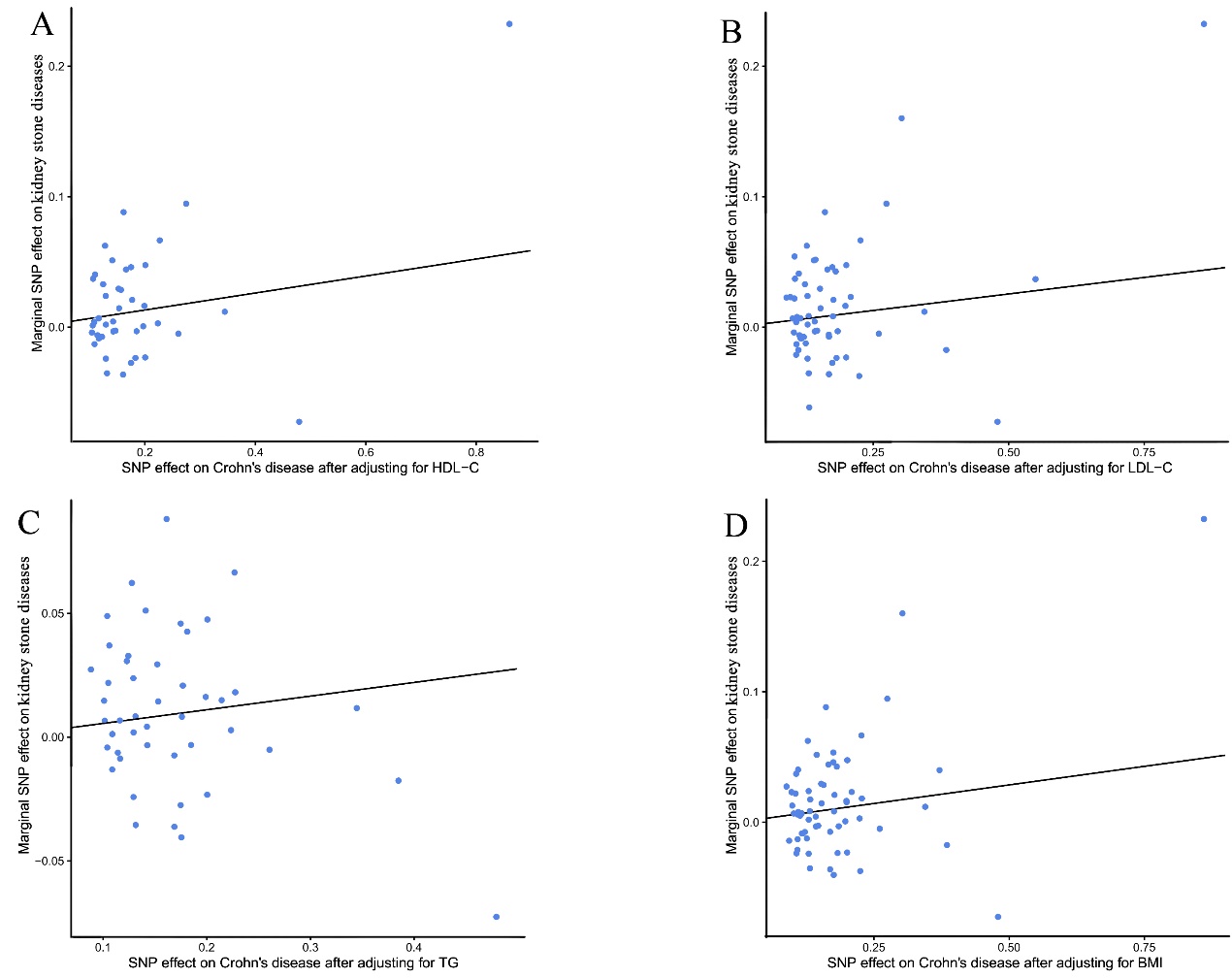


MVMR, multivariable mendelian randomization; SNP, single-nucleotide polymorphism; CD, Crohn’s disease; KSD, kidney stone disease; HDL-C, high-density lipoprotein cholesterol; LDL-C, low-density lipoprotein cholesterol; TG, triglycerides; BMI, body mass index

**Figure S11.** Scatter plots of the degree of effect of each SNP on UC and KSD in MVMR. (A) Adjusting for HDL-C; (B) Adjusting for LDL-C; (C) Adjusting for TG; (D) Adjusting for BMI.


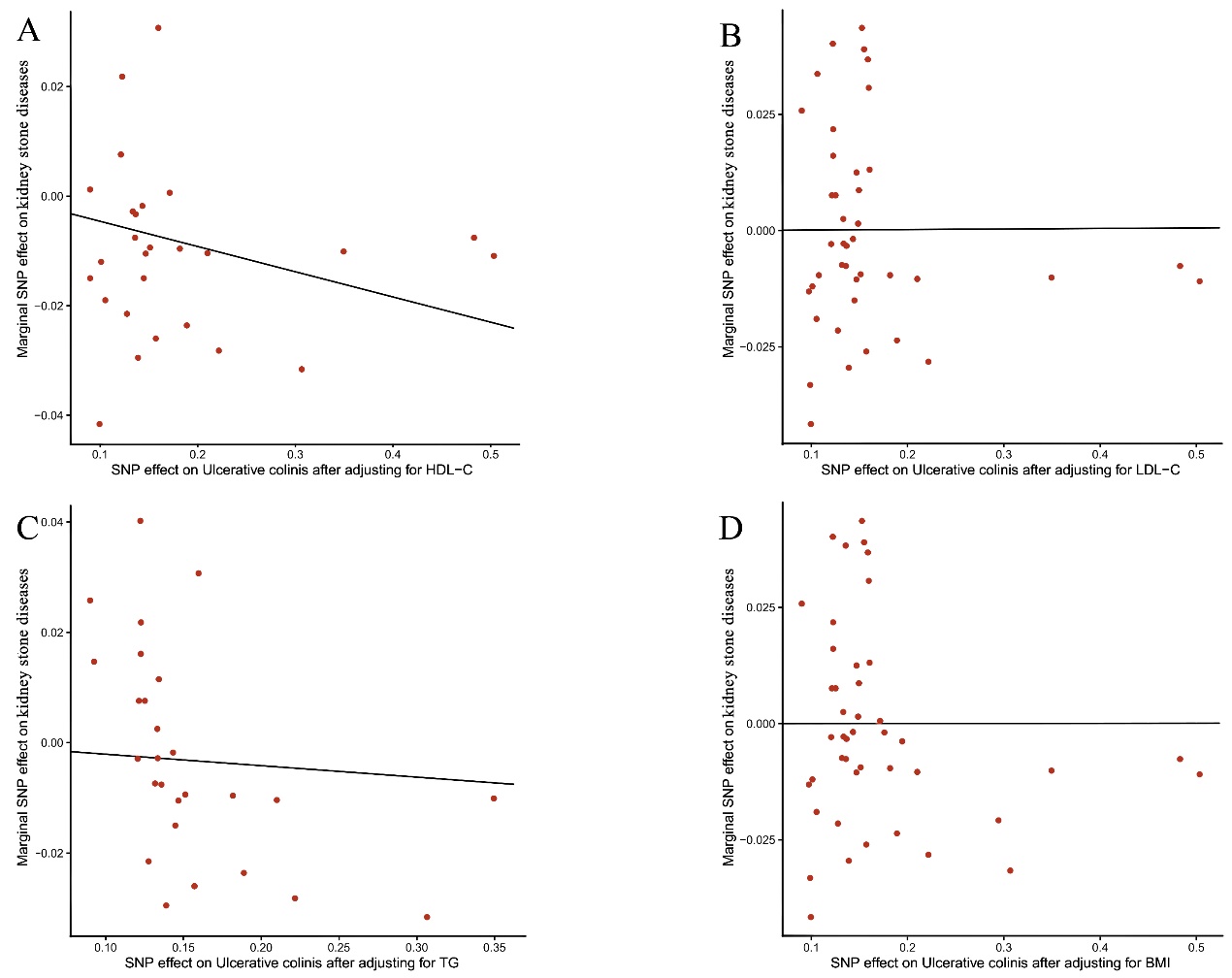


MVMR, multivariable mendelian randomization; SNP, single-nucleotide polymorphism; UC, ulcerative colitis; KSD, kidney stone disease; HDL-C, high-density lipoprotein cholesterol; LDL-C, low-density lipoprotein cholesterol; TG, triglycerides; BMI, body mass index
